# Persistent Dirac for molecular representation

**DOI:** 10.1038/s41598-023-37853-z

**Published:** 2023-07-11

**Authors:** Junjie Wee, Ginestra Bianconi, Kelin Xia

**Affiliations:** 1 Division of Mathematical Sciences, School of Physical and Mathematical Sciences, Nanyang Technological University, Singapore, 637371 Singapore; 2 School of Mathematical Sciences, Queen Mary University of London, London, E1 4NS UK; 3 The Alan Turing Institute, London, NW1 2DB UK

**Keywords:** Solar cells, Machine learning, Computational science

## Abstract

Molecular representations are of fundamental importance for the modeling and analysing molecular systems. The successes in drug design and materials discovery have been greatly contributed by molecular representation models. In this paper, we present a computational framework for molecular representation that is mathematically rigorous and based on the persistent Dirac operator. The properties of the discrete weighted and unweighted Dirac matrix are systematically discussed, and the biological meanings of both homological and non-homological eigenvectors are studied. We also evaluate the impact of various weighting schemes on the weighted Dirac matrix. Additionally, a set of physical persistent attributes that characterize the persistence and variation of spectrum properties of Dirac matrices during a filtration process is proposed to be molecular fingerprints. Our persistent attributes are used to classify molecular configurations of nine different types of organic-inorganic halide perovskites. The combination of persistent attributes with gradient boosting tree model has achieved great success in molecular solvation free energy prediction. The results show that our model is effective in characterizing the molecular structures, demonstrating the power of our molecular representation and featurization approach.

## Introduction

Molecular representation and featurization play an essential role in physical as well as in data-driven learning models. The relationship between the structure and function of molecules is complex, and a comprehensive understanding of the structural properties is crucial to extract functional information. To establish explicit linear or nonlinear relationships between molecular structure and function, various quantitative structure-activity/property relationship (QSAR/QSPR) models have been developed^1,2^. Different molecular fingerprints have also been proposed for machine learning and deep learning models to predict molecular functions and properties^3–8^. Despite significant advances, the development of highly efficient descriptors remains a major challenge for QSAR/QSPR and learning models in the analysis of molecular data in the fields of materials, chemistry, and biology^1,2^.

Graph models^9–18^ are arguably the most widely used tools for molecular representations in molecular dynamics simulation, coarse-grained models, elastic network models, QSAR/QSPR, graph neural networks, etc. In general, a molecule (or a molecular complex) is modeled as a graph with each vertex representing an atom, an amino acid, a domain, or an entire molecule, and edge representing covalent-bond, non-covalent-bond, or more general interaction. However, graphs are designed for the characterization of pairwise interactions. To capture higher-order interactions, topological representations, such as multilayer networks^19^, simplicial complexes^20–22^, hypergraphs^23,24^, etc, should be considered. Among them, multilayer networks have been used in the characterization of higher-order dynamics^25–28^ and synchronization dynamics^29–31^. As a generalization of graphs, simplicial complexes are made not only of 0-simplices (nodes) and 1-simplices (edges), but also of higher-dimensional simplices, such as 2-simplices (triangles), 3-simplices (tetrahedron), etc. Note that higher-order networks and simplicial complexes can describe the many-body interactions beyond pairwise interactions. Hypergraphs are a further generalization of simplicial complexes. An hypergraph is composed of hyperedges, which are formed by a set of vertices. Recently, simplicial complexes and hypergraphs have been used in molecular representations and have allowed improved performance of drug design algorithms, in particular, in the protein-ligand binding affinity prediction.

Based on topological representations, molecular descriptors or fingerprints can be generated and further used as features for learning models. The recent emergence of topological data analysis (TDA)^32,33^ and combinatorial Hodge theory-based molecular descriptors has had a significant impact on drug design. These models have been successful in various stages of drug design, such as predicting protein-ligand binding affinity^4,14,34–39^, protein stability changes resulting from mutations^40,41^, toxicity^42^, solvation free energy^43,44^, partition coefficient and aqueous solubility^45^, and identifying binding pockets^46^. In comparison to traditional molecular representations, these models have demonstrated superior performance in the D3R Grand Challenge^47,48^. TDA’s fundamental mathematical concept involves using persistent homology to extract topological information, tracking the change of homology generators of simplicial complexes over a filtration process. In particular, the topological invariant Betti numbers can be obtained from the kernel of combinatorial Hodge Laplacian (HL) matrix. Interestingly, the Forman Ricci curvature can be obtained via the Bochner–Weitzenböck decomposition of HL matrix^6^. The great success of TDA and combinatorial Hodge theory based molecular descriptors in learning models is due to their characterization of structures with intrinsic invariants, including Betti numbers and Ricci curvatures. These intrinsic descriptors are well defined mathematical observables that characterize fundamental topological and geometrical properties of real datasets, thus they have an excellent transferability for learning models.

Inspired by the success of Hodge Laplacian matrix in molecular sciences, here we propose the persistent Dirac based molecular representation and fingerprint. The discrete Dirac operator^49–55^ is a first-order differential operator which can be interpreted as the square root of Hodge Laplace operator. This operator has been developed on graphs and simplicial complexes and used in TDA and for investigating dynamics of topological signals^50,56–58^. Moreover, the persistent Dirac model can be used in the quantum algorithm of persistent homology^52,53,59^. Here we present a rigorous mathematical theory for persistent Dirac through the commutative diagram of discrete Dirac operator over a filtration process. The commutative diagram is similar to the ones in persistent spectral graph^5,60^, persistent Hodge Laplacian^61^, and persistent sheaf Laplacian^61,62^. Further, we develop a series of persistent attributes from persistent Dirac, and use them as descriptors to characterize molecular structures.

Our work starts with a systematic study of the spectrum of the discrete Dirac matrix. In particular, we identify the geometric and topological properties of both non-homology and homology eigenvectors for molecular structures. We generalize these results to weighted simplicial complexes on top of which the weighted Dirac operator^63^ is carefully defined. In particular, we analyse the influence of weighting schemes on the spectral properties of molecular structures. The persistent Dirac is then introduced and is employed for the clustering of molecular configurations from the molecular dynamic simulations of nine types of organic-inorganic halide perovskites (OIHP). By the comparison with several existing models, we show that our model is highly efficient in clustering the structure configurations. Further, the combination of persistent attributes with gradient boosting tree model has achieved great success in molecular solvation free energy prediction. This demonstrates the great potential of our persistent Dirac-based fingerprints in molecular representation and featurization.

The paper is organized as follows. Section “Methods” is devoted to the discrete Dirac models. It covers basic mathematical background such as simplicial complexes, chain groups, boundary operators, Hodge Laplacian. Thereafter, the section discusses the use of spectrum of discrete Dirac models for biomolecular representation and characterization. In section “Results”, persistent Dirac model is present. The eigenspectrum information for (weighted) Dirac matrix and persistent attributes from persistent Dirac are discussed in detailed. The section ends with an application of the persistent Dirac based fingerprints on organic-inorganic halide perovskite (OIHP) classification and prediction of solvation free energy. The paper ends with a conclusion.

## Methods

In this section, we discuss the discrete Dirac models, including discrete Dirac matrices and weighted Dirac matrices for biomolecular structure representation and characterization. Different from previous graph-based models, molecular structures are represented based on simplicial complexes, and algebraic tools from chain groups, homology groups, boundary operators and Dirac matrices, are used to reveal deeper geometric and topological properties.

### Mathematical background for discrete Dirac models

#### Simplicial complex

Generally speaking, a simplicial complex can be viewed as a higher-dimensional generalization of graphs. A *p*-dimensional simplicial complex is formed by simplices of dimension up to *p*. Every *p* dimensional simplex consists of a set of $p+1$ vertices and this set can be viewed geometrically as a point (0-simplex), an edge (1-simplex), a triangle (2-simplex), a tetrahedron (3-simplex), etc.

Here and in the following we indicate with $n_p$ the number of *p*-simplices belonging to the simplicial complex ${\mathcal {K}}$. The most commonly used simplicial complexes include Čech complex, Vietoris–Rips complex, Alpha complex, Cubical complex, Morse complex, etc.^64^.

Two *p*-dimensional simplices $\sigma _1$ and $\sigma _2$ in a simplicial complex ${\mathcal {K}}$, are simplex neighbors if

(i) $\sigma _1$ and $\sigma _2$ share a $(p+1)$-simplex $\mu$, that is, there exists a $\mu$ in ${\mathcal {K}}$ such that $\mu > \sigma _1$ and $\mu >\sigma _2$.
(ii) $\sigma _1$ and $\sigma _2$ share a $(p-1)$-simplex $\gamma$, that is, there exists a $\gamma$ in ${\mathcal {K}}$ such that $\gamma < \sigma _1$ and $\gamma < \sigma _2$.

If either condition is satisfied, but both conditions do not hold at the same time, $\sigma _1$ and $\sigma _2$ are called parallel simplex neighbors. Here $\sigma _1$ and $\sigma _2$ are called upper adjacent neighbors and denoted as $\sigma _1 \frown \sigma _2$, if they satisfy condition (i). They are lower adjacent neighbors and denoted as $\sigma _1 \smile \sigma _2$ if they satisfy condition (ii).

#### Homology

In homology, a *p*-dimensional oriented simplex $\sigma ^p$ is the set of ordered $p+1$ nodes $[v_0, v_1, \ldots , v_p]$. For example, an oriented 1-simplex $\sigma ^1 =[v_0, v_1]$ has the opposite sign of the oriented 1-simplex $[v_1, v_0]$. In other words,

$$[v_i, v_j] = -[v_j, v_i].$$

Similarly, this orientation can be written for higher-order simplices in the following way,

$$[v_0, v_1, \ldots , v_p] = (-1)^{\alpha (\pi )} [v_{\pi (0)}, v_{\pi (1)}, \ldots , v_{\pi (p)}],$$

where $\alpha (\pi )$ refers to the parity of the permutation $\pi$. In this paper, we consider the orientation induced by node labels, i.e. for every simplex in a simplicial complex, we assign a positive orientation to the one provided by the increasing set of node labels.

For an oriented simplicial complex ${\mathcal {K}}$, its *p*-dimensional chain group $C_p({\mathcal {K}})$ is composed by linear combination of positively oriented *p*-simplices in ${\mathcal {K}}$. Let $[v_0, v_1, \ldots v_p]$ indicate the generic positively oriented *p*-simplex $\sigma ^p\in {\mathcal {K}}$. We notice that the set of simplices $\sigma _p$ constitute a basis for the *p*-dimensional chains $C_p({\mathcal {K}})$. Therefore any *p*-chain $f_1\in C_p({\mathcal {K}})$ can be written in a unique way as

1 $$f_1=\sum _{i=1}^{n_p}c_i\sigma ^i.$$

The weighted boundary operator $\overline{\partial }_p: C_p \rightarrow C_{p-1}$ can be determined by its action on any given $\sigma ^p \in {\mathcal {K}}$:

$$\overline{\partial }_p(\sigma ^p) = a_p\sum _{i=0}^p (-1)^i [v_0, v_1, \ldots , \hat{v}_i, \ldots , v_p].$$

Here $a_p$ is a constant in ${\mathbb {R}}^+$ dependent on *p* and the boundary of *p*-simplex is made of $(p-1)$-simplices $[v_0, v_1, \ldots , \hat{v}_i, \ldots , v_p]$, where $\hat{v_i}$ means that $v_i$ has been removed from the sequence $v_0, \ldots , v_p$. It is also well-known that $\overline{\partial }_{p-1}\overline{\partial }_{p} = 0$. The unweighted boundary operator can be obtained by setting $a_p=1$. In other words, the unweighted boundary operator $\partial _p: C_p \rightarrow C_{p-1}$ for a given $\sigma ^p \in {\mathcal {K}}$ is defined as

$$\begin{aligned} \partial _p(\sigma ^p) = \sum _{i=0}^p (-1)^i [v_0, v_1, \ldots , \hat{v}_i, \ldots , v_p]. \end{aligned}$$

For an oriented simplicial complex ${\mathcal {K}}$, its two oriented *p*-dimensional simplices $\sigma _1$ and $\sigma _2$ are similarly oriented and denoted as $\sigma _1 \sim \sigma _2$, if they are lower adjacent and have the same sign on the common lower $(p-1)$-simplex. Two simplex $\sigma _1$ and $\sigma _2$ are dissimilarly oriented and denoted as $\sigma _1 \nsim \sigma _2$, if they are lower adjacent but have different signs on the common lower $(p-1)$-simplex.

The *p*-th cycle group $Z_p$ is defined as,

$$\begin{aligned} Z_p ={\text {ker}}(\overline{\partial }_{p}) = \{c \in C_p | \overline{\partial }_{p}(c) = 0\}, \end{aligned}$$

and *p*-th boundary group $B_p$ is,

$$\begin{aligned} B_p ={\text {im}}(\overline{\partial }_{p+1}) = \{c\in C_p | \exists d \in C_{p+1}: c = \overline{\partial }_{p+1}(d) \}. \end{aligned}$$

The *p*-th homology group is defined as $H_p = Z_p/B_p$. Its rank is *p*-th Betti number that satisfies

$$\begin{aligned} \beta _p = \text {rank } H_p = \text {rank } Z_p - \text {rank } B_p. \end{aligned}$$

With the boundary operators, we have chain complexes

$$\begin{aligned} \cdots \xrightarrow {\overline{\partial }_{p+2}} C_{p+1}\xrightarrow {\overline{\partial }_{p+1}} C_{p}\xrightarrow {\overline{\partial }_{p}} C_{p-1}\xrightarrow {\overline{\partial }_{p-1}} \cdots \end{aligned}$$

The adjoint of $\overline{\partial }_p$, which is

$$\begin{aligned} \overline{\partial }_{p}^*:C_{p-1}\rightarrow C_{p}, \end{aligned}$$

satisfies the inner product relation $\langle \overline{\partial }_p(f), g \rangle =\langle f, \overline{\partial }_{p}^*(g) \rangle ,$ for every $f \in C_{p}$, $g \in C_{p-1}$. It is used in the weighted Hodge Laplacian.

#### Weighted Hodge Laplacian and Hodge decomposition

The *p*-dimensional weighted Hodge Laplacian $\Delta _p : C_p \rightarrow C_p$ is defined as follows:

$$\begin{aligned} \Delta _p = {\left\{ \begin{array}{ll} \overline{\partial }_1\circ \overline{\partial }_1^*, &\quad \text {if } p=0.\\ \overline{\partial }_{p}^*\circ \overline{\partial }_{p} + \overline{\partial }_{p+1}\circ \overline{\partial }_{p+1}^*, &\quad \text {if } p\ge 1. \end{array}\right. } \end{aligned}$$

The special case where $p = 0$ is the well-known graph Laplacian.

Computationally, the information for weighted boundary operators acting from finite dimensional chain groups $C_p$ to $C_{p-1}$ can be stored efficiently in matrix representations. As matrix representations, the weighted boundary operators and its adjoint satisfies $\overline{\partial }_{p}^{\top} = \overline{\partial }_{p}^*$.

More specifically, let $n_{p-1}$ and $n_p$ be the number of $(p-1)$-simplices and *p*-simplices respectively in a simplicial complex ${\mathcal {K}}$. The $n_{p-1}\times n_p$ weighted boundary matrix $\overline{\textbf{B}}_p$ has entries defined as follows:

$$\begin{aligned} \overline{\textbf{B}}_p (i,j) = \left\{ \begin{array}{ll} a_p, &\quad \text {if } \sigma _i^{p-1}< \sigma _j^p, \sigma _i^{p-1} \sim \sigma _j^p.\\ -a_p, &\quad \text {if } \sigma _i^{p-1} < \sigma _j^p, \sigma _i^{p-1} \nsim \sigma _j^p.\\ 0, &\quad \text {if } \sigma _i^{p-1} \nless \sigma _j^p. \end{array} \right. \end{aligned}$$

where $1\le i\le n_{p-1}$ and $1\le j\le n_p$. Here, $\sigma _i^{p-1} < \sigma _j^p$ represents the *i*-th $(p-1)$-simplex $\sigma _i^{p-1}$ is a face of *j*-th *p*-simplex $\sigma _j^p$ and $\sigma _i^{p-1} \sim \sigma _j^p$ indicates the coefficient of $\sigma _i^{p-1}$ in $\overline{\partial }_{p}(\sigma _j^p)$ is $a_p$. Likewise, $\sigma _i^{p-1} \nless \sigma _j^p$ means that $\sigma _i^{p-1}$ is not a face of $\sigma _j^p$ and $\sigma _i^{p-1} \nsim \sigma _j^p$ indicates that the coefficient of $\sigma _i^{p-1}$ in $\overline{\partial }_{p}(\sigma _j^p)$ is $-a_p$.

Since the unweighted boundary operator $\partial _{p}=\frac{1}{a_p}\overline{\partial }_p$, note that an unweighted boundary matrix can be similarly written as

2 $$\textbf{B}_p =\frac{1}{a_p}\overline{\textbf{B}}_p.$$

Using the weighted boundary matrices, the lower and upper weighted Hodge Laplacians can be defined as $\overline{\textbf{L}}_p^{{\text {down}}} = \overline{\textbf{B}}_p^{{\top}} \overline{\textbf{B}}_p$ and $\overline{\textbf{L}}_p^{{\text {up}}} = \overline{\textbf{B}}_{p+1}\overline{\textbf{B}}_{p+1}^{{\top}}$ respectively. More specifically, the entries of $\overline{\textbf{L}}_p^{{\text {down}}}$ ($p>0$) are as follows,

$$\overline{\textbf{L}}_{p}^{{\text {down}}} (i,j) = \left\{ \begin{array}{ll} a_{p}^{2}(p+1), & i=j. \\ a_{p}^{2}, & i\ne j, \sigma ^{p}_{i} \smile \sigma ^{p}_{j}, \sigma ^{p}_{i} \sim \sigma ^{p}_{j}. \\ -a_{p}^{2}, & i\ne j, \sigma ^{p}_{i} \smile \sigma ^{p}_{j}, \sigma ^{p}_{i} { {\nsim }} \sigma ^{p}_{j}. \\ 0, & i\ne j \text{ and } \sigma ^{p}_{i} {{\not \smile}} \sigma ^{p}_{j}. \end{array} \right.$$

Note that all the entries of $\overline{\textbf{L}}_{0}^{{\text {down}}}$ are zero since 0-simplices have no lower adjacent neighbors. Further, $\sigma ^{p}_{i} \smile \sigma ^{p}_j$ refers to $\sigma ^{p}_{i}$ and $\sigma ^{p}_{j}$ being lower adjacent neighbors while $\sigma ^{p}_{i} \frown \sigma ^{p}_{j}$ refers to $\sigma ^{p}_{i}$ and $\sigma ^{p}_{j}$ being upper adjacent neighbors.

It is important to observe that $\sigma ^{p}_{i} \frown \sigma ^{p}_{j}$ $(p>0)$ also implies that $\sigma ^{p}_i$ and $\sigma ^{p}_j$ share a lower simplex $\sigma ^{p-1}$. The case where $\sigma ^{p}_i \sim \sigma ^{p}_j$ refers to $\sigma ^{p}_i$ and $\sigma ^{p}_j$ sharing a common similar lower simplex $\sigma ^{p-1}$. This means that the signs of coefficient of $\sigma ^{p-1}$ in $\overline{\partial }_{p}(\sigma _i^{p})$ and $\overline{\partial }_{p}(\sigma _j^{p})$ are the same. On the other hand, $\sigma ^{p}_{i} { {\nsim }} \sigma ^{p}_j$ refers to $\sigma ^{p}_{i}$ and $\sigma ^{p}_j$ sharing a common dissimilar lower simplex $\sigma ^{p-1}$. This can be verified by checking the signs of coefficient of $\sigma ^{p-1}$ in $\overline{\partial }_{p}(\sigma _i^{p})$ and $\overline{\partial }_{p}(\sigma _j^{p})$ to be not the same.

Since 0-simplices have no lower adjacent neighbors, any two 0-simplices $\sigma ^{0}_i$ and $\sigma ^{0}_j$ that are upper adjacent neighbors will always satisfy $\sigma ^{0}_i \sim \sigma ^{0}_j$ vacuously.

Hence, the matrix elements of the Hodge Laplacian $\overline{\textbf{L}}_p^{\text {up}}$ are given by

$$\overline{\textbf{L}}_{p}^{\text {up}} (i,j) = \left\{ \begin{array}{ll} a_{p+1}^{2} d(\sigma ^{p}_{i}), & i=j. \\ -a_{p+1}^{2}, & i\ne j, \sigma ^{p}_{i} \frown \sigma ^{p}_{j}, \sigma ^{p}_{i} \sim \sigma ^{p}_{j}. \\ a_{p+1}^{2}, & i\ne j, \sigma ^{p}_{i} \frown \sigma ^{p}_{j}, \sigma ^{p}_{i} \nsim \sigma ^{p}_{j}. \\ 0, & i\ne j \text { and } \sigma ^{p}_{i} \not \frown \sigma ^{p}_{j}. \end{array} \right.$$

Here $d(\sigma ^{p}_i)$ denotes the number of cofaces with dimension $p+1$ of simplex $\sigma ^{p}_i$.

The $p^{\text {th}}$ weighted combinatorial Laplacian $\overline{\textbf{L}}_p$ is defined as $\overline{\textbf{L}}_p=\overline{\textbf{B}}_p^{\top} \overline{\textbf{B}}_p + \overline{\textbf{B}}_{p+1}\overline{\textbf{B}}_{p+1}^{\top}$. Note that $\overline{\textbf{L}}_0=\overline{\textbf{B}}_1\overline{\textbf{B}}_1^{\top}$. The matrix elements of the Hodge Laplacians $\overline{\textbf{L}}_p$ with $p=0$ are given by

$$\overline{\textbf{L}}_{0} (i,j) = \left\{ \begin{array}{ll} a_{1}^{2}d(\sigma ^{0}_{i}), & i=j. \\ -a_{1}^{2}, & i\ne j, \sigma ^{0}_{i} \frown \sigma ^{0}_{j}.\\ 0, & i\ne j, \sigma ^{0}_{i} \not \frown \sigma ^{0}_{j}. \end{array} \right.$$

while the matrix elements for $p>0$ can be expressed as

$$\overline{\textbf{L}}_{p} (i,j)=\left\{ \begin{array}{ll} a_{p+1}^{2}d(\sigma ^{p}_{i})+a_{p}^{2}(p+1), & i=j. \\ a_{p}^{2} - a_{p+1}^{2}, & i\ne j, \sigma ^{p}_{i} \frown \sigma ^{p}_{j}, \sigma ^{p}_{i} \smile \sigma ^{p}_{j}, \sigma ^{p}_{i} \sim \sigma ^{p}_{j}. \\ a_{p+1}^{2} - a_{p}^{2}, & i\ne j, \sigma ^{p}_{i} \frown \sigma ^{p}_{j}, \sigma ^{p}_{i} \smile \sigma ^{p}_{j}, \sigma ^{p}_{i} \nsim \sigma ^{p}_{j}. \\ a_{p}^{2}, & i\ne j, \sigma ^{p}_{i} \not \frown \sigma ^{p}_{j}, \sigma ^{p}_{i} \smile \sigma ^{p}_{j}, \sigma ^{p}_{i} \sim \sigma ^{p}_{j}. \\ -a_{p}^{2}, & i\ne j, \sigma ^{p}_{i} \not \frown \sigma ^{p}_{j}, \sigma ^{p}_{i} \smile \sigma ^{p}_{j}, \sigma ^{p}_{i} \nsim \sigma ^{p}_{j}. \\ 0, & i\ne j \text { and }\sigma ^{p}_{i} \not \smile \sigma ^{p}_{j}. \end{array} \right.$$

It follows from Eq. (2) that the lower and upper unweighted Hodge Laplacians can be written as $\textbf{L}_{p}^{\text {down}} = \textbf{B}_{p}^{\top} \textbf{B}_{p}$ and $\textbf{L}_{p}^{\text {up}} = \textbf{B}_{p+1}\textbf{B}_{p+1}^{\top}$ respectively. Hence, the $p{\text {th}}$ unweighted combinatorial Laplacian $\textbf{L}_{p} = \textbf{L}_{p}^{\text {down}} + \textbf{L}_{p}^{\text {up}}$ have elements given by

$$\textbf{L}_0 (i,j) = \left\{ \begin{array}{ll} d(\sigma ^{0}_{i}), & i=j. \\ -1, & i\ne j, \sigma ^{0}_{i} \frown \sigma ^{0}_{j}.\\ 0, & i\ne j, \sigma ^{0}_{i} \not \frown \sigma ^{0}_{j}. \end{array} \right.$$

for $p=0$ while for $p>0$ the matrix elements of the Hodge Laplacian are given by

$$\textbf{L}_p (i,j)=\left\{ \begin{array}{ll} d(\sigma ^{p}_{i})+p+1, & i=j. \\ 1, & i\ne j, \sigma ^{p}_{i} \not \frown \sigma ^{p}_{j}, \sigma ^{p}_{i} \smile \sigma ^{p}_{j}, \sigma ^{p}_{i} \sim \sigma ^{p}_{j}. \\ -1, & i\ne j, \sigma ^{p}_{i} \not \frown \sigma ^{p}_{j}, \sigma ^{p}_{i} \smile \sigma ^{p}_{j}, \sigma ^{p}_{i} \nsim \sigma ^{p}_{j}. \\ 0, & i\ne j \text { and either } \sigma ^{p}_{i} \frown \sigma ^{p}_{j} \text { or } \sigma ^{p}_{i} \not \smile \sigma ^{p}_{j}. \end{array} \right.$$

It is well-known that $\lambda$ is a non-zero eigenvalue of $\overline{\textbf{L}}_p$ if and only if $\lambda$ is an non-zero eigenvalue of $\overline{\textbf{L}}_p^{\text {down}}$ or $\overline{\textbf{L}}_p^{\text {up}}$. The multiplicity of the zero eigenvalues of $\overline{\textbf{L}}_p$ corresponds to the *p*th Betti number as follows,

$$\begin{aligned} \dim \ker \overline{\textbf{L}}_p = \beta _p = \dim \ker \overline{\textbf{L}}_p^{\text {down}} - \dim {\text {im}} \overline{\textbf{L}}_p^{\text {up}} \end{aligned}$$

where $\beta _p$ is also the $\text {rank }H_p$^65^ (see Appendix C).

Further, $\dim \ker \overline{\textbf{L}}_p^{\text {down}}$ can be written as:

3 $$\begin{aligned} \dim \ker \overline{\textbf{L}}_p^{\text {down}}&= \beta _p + \dim {\text {im}} \overline{\textbf{L}}_p^{\text {up}}\nonumber \\&= \beta _p + \dim C_p - \dim \ker \overline{\textbf{L}}_p^{\text {up}}\nonumber \\&= \beta _p + \dim C_p - \dim \ker \overline{\textbf{B}}_{p+1}^{\top} \nonumber \\&= \beta _p + \text {rank } \overline{\textbf{B}}_{p+1}^{\top} . \end{aligned}$$

The above Eq. (3) will be an important relation for persistent Dirac models in later sections.

Closely related to the Hodge Laplacian is the Hodge decomposition. The Hodge decomposition is an orthogonal decomposition of a vector field into gradient part, harmonic part and curl part. More formally, the Hodge decomposition states that a *p*-th chain group $C_p$ of a simplicial complex ${\mathcal {K}}$ admits the following orthogonal direct sum decomposition:
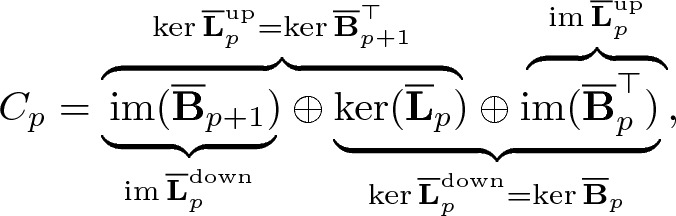


where $$\ker (\overline{\textbf{L}}_p) = \ker \overline{\textbf{B}}_p \cap \ker \overline{\textbf{B}}_{p+1}^{\top}$$.

It is worth mentioning that such flows have also been extended to five component decompositions with edge and face vector fields^66^, applied to the protein B-factor prediction problems via Hodge theory^8^ and also in de Rham–Hodge biomolecular data analysis^9,67^.

### Discrete Dirac models

#### Weighted Dirac matrix

Recently, weighted Dirac matrices have been proposed based on a weighted simplicial complex^63^. For a *d*-dimensional weighted simplicial complex ${\mathcal {K}}$, let us define the $n_{p} \times n_{p}$ metric matrix $\textbf{G}_{p}$ ($0\le p \le d$) to be a diagonal matrix with positive entries. For any two *p*-chains $f_1=\sum _{i=1}^{n_p}c_i\sigma ^i$ and $f_2=\sum _{i=0}^{n_p}d_i\sigma ^i$ in $C_p$, the matrix $\textbf{G}_{p}$ can be used to define the weighted inner product

4 $$\left\langle f_1, f_2\right\rangle = \sum _{i=1}^{n_{p}} \textbf{G}_{p}(\sigma ^{i},\sigma ^{i})c_{i}d_i = (\textbf{f}_1)^{\top} \textbf{G}_{p}(\textbf{f}_2),$$

where $$(\textbf{f}_1)^{\top} =[c_1, c_2, \ldots , c_p]$$ and $$(\textbf{f}_2)^{\top} =[d_1, d_2, \ldots , d_p]$$.

Recall from Eq. (2) that the weighted boundary operator can be represented by a matrix $\overline{\textbf{B}}_p =a_p\textbf{B}_p$ where $\textbf{B}_p$ is the unweighted boundary matrix. If $a_p=1$, then $\overline{\textbf{B}}_p$ reduces to the adjoint operator of $\textbf{B}_p$. Formally, for any *p*-chain *f* and any $(p-1)$-chain *g*, the adjoint operator $\overline{\textbf{B}}_p^*$ satisfies

5 $$\langle f, \overline{\textbf{B}}_p^*g \rangle = \langle \overline{\textbf{B}}_pf, g\rangle.$$

From the inner product relation (5), an explicit expression of $\overline{\textbf{B}}_p^*$ can be deduced in terms of $\overline{\textbf{B}}_p$ and the matrices $\textbf{G}_p$. Based on the weighted inner product definition (4), this gives

$$\begin{aligned} (\textbf{f})^{\top} \textbf{G}_p\overline{\textbf{B}}_p^*(\textbf{g}) = (\textbf{f})^{\top} \overline{\textbf{B}}_p^{\top} \textbf{G}_{p-1}(\textbf{g}). \end{aligned}$$

Since the expression is true for any arbitrary $\textbf{f}$ and $\textbf{g}$, this implies

$$\begin{aligned} \textbf{G}_p\overline{\textbf{B}}_p^* = \overline{\textbf{B}}_p^{\top} \textbf{G}_{p-1}. \end{aligned}$$

Hence, the following becomes an explicit expression for the adjoint operator $\overline{\textbf{B}}_p^*$:

6 $$\begin{aligned} \overline{\textbf{B}}_p^* = \textbf{G}_{p}^{-1} \overline{\textbf{B}}_p^{\top} \textbf{G}_{p-1}. \end{aligned}$$

Here $$\overline{\textbf{B}}_p^*$$^65^ is the adjoint of the weighted boundary operator^10,68^. It is important to note that if the metric matrices $$\textbf{G}_p$$ are the identity matrices, the above expression then reduces to the transpose of the boundary operator multiplied by the constant $$a_p$$,

7 $$\begin{aligned} \overline{\textbf{B}}_p^* = 
\overline{\textbf{B}}_p^{\top} = a_p\textbf{B}_p^{\top} . \end{aligned}$$

This also means the transpose of adjoint operator, i.e. $(\overline{\textbf{B}}_p^*)^{\top}$, is equal to $\overline{\textbf{B}}_p$ only if $\textbf{G}_p$ are identity matrices. To see this, apply the transpose to both sides of Eq. (6) and obtain the expression

8 $$\begin{aligned} (\overline{\textbf{B}}_p^*)^{\top} = \textbf{G}_{p-1}^{-1}\overline{\textbf{B}}_p\textbf{G}_{p}=a_p\textbf{G}_{p-1}^{-1}\textbf{B}_p\textbf{G}_{p}. \end{aligned}$$

The matrices $(\overline{\textbf{B}}_p^*)^{\top}$ and $\overline{\textbf{B}}_p^*$ can then be used to construct the following weighted Dirac matrix (9).

For a simplicial complex ${\mathcal {K}}$ with $n_{p} \times n_{p-1}$ adjoint operators $\overline{\textbf{B}}_p^*$ where $n_{p-1}$ is the number of $(p-1)$-simplices and $n_p$ is the number of *p*-simplices in ${\mathcal {K}}$, the weighted Dirac matrix $\overline{\textbf{D}}_p$ is

9 $$\begin{aligned} \overline{\textbf{D}}_p= \begin{bmatrix} \text{0 }_{n_0\times n_0} &\quad (\overline{\textbf{B}}_1^*)^{\top} &\quad \text{0 }_{n_0\times n_2} &\quad \cdots &\quad \text{0 }_{n_0\times n_{p}} &\quad \text{0 }_{n_0\times n_{p+1}} \\ \overline{\textbf{B}}_1^* &\quad \text{0 }_{n_1\times n_1} &\quad (\overline{\textbf{B}}_2^*)^{\top} &\quad \cdots &\quad \text{0 }_{n_1\times n_{p}} &\quad \text{0 }_{n_1\times n_{p+1}} \\ \text{0 }_{n_2\times n_0} &\quad \overline{\textbf{B}}_2^* &\quad \text{0 }_{n_2\times n_2} &\quad \cdots &\quad \text{0 }_{n_2\times n_{p}} &\quad \text{0 }_{n_2\times n_{p+1}} \\ \vdots &\quad \vdots &\quad \vdots &\quad \vdots &\quad \vdots &\quad \vdots \\ \text{0 }_{n_{p}\times n_0} &\quad \text{0 }_{n_{p}\times n_1} &\quad \text{0 }_{n_{p}\times n_2} &\quad \cdots &\quad \text{0 }_{n_{p} \times n_{p}} &\quad (\overline{\textbf{B}}_{p+1}^*)^{\top} \\ \text{0 }_{n_{p+1}\times n_0} &\quad \text{0 }_{n_{p+1}\times n_1} &\quad \text{0 }_{n_{p+1}\times n_2} &\quad \cdots &\quad \overline{\textbf{B}}_{p+1}^* &\quad \text{0 }_{n_{p+1}\times n_{p+1}} \end{bmatrix}. \end{aligned}$$

In particular, we set $a_p=(p+1)^{-1/2}$ for all *p* up to the order of the simplicial complex and consider the matrices $\overline{\textbf{B}}_p^*$ and $(\overline{\textbf{B}}_p^*)^{\top}$ in Eq. (7) and (8) respectively. For $p=2$, the weighted Dirac matrix from (9) becomes

$$\begin{aligned} \overline{\textbf{D}}_1=\begin{bmatrix} \text{0 }_{n_0\times n_0} &\quad \textbf{G}_{0}^{-1}\textbf{B}_1\textbf{G}_{1}/\sqrt{2} &\quad \text{0 }_{n_0\times n_2} &\quad \text{0 }_{n_0\times n_{3}}\\ \textbf{B}_1^{\top} /\sqrt{2} &\quad \text{0 }_{n_1\times n_1} &\quad \textbf{G}_{1}^{-1}\textbf{B}_2\textbf{G}_{2}/\sqrt{3} &\quad \text{0 }_{n_1\times n_{3}}\\ \text{0 }_{n_2\times n_0} &\quad \textbf{B}_2^{\top} /\sqrt{3} &\quad \text{0 }_{n_2\times n_2} &\quad \textbf{G}_{2}^{-1}\textbf{B}_3\textbf{G}_{3}/2\\ \text{0 }_{n_3\times n_0} &\quad \text{0 }_{n_3\times n_1} &\quad \textbf{B}_3^{\top} /2 &\quad \text{0 }_{n_3\times n_3} \end{bmatrix} \end{aligned}$$

This definition can be extended easily to higher dimensions. Note that $\textbf{B}_p^{\top} /\sqrt{p+1}$ is the adjoint operator $\overline{\textbf{B}}_p^*$ and $\textbf{G}_{p-1}^{-1}\textbf{B}_p\textbf{G}_{p}/\sqrt{p+1}$ is equal to the transpose of $\overline{\textbf{B}}_p^*$. Note that this definition of weighted Dirac is self-adjoint and with eigenvalues smaller than or equal to one. The square of the weighted Dirac also forms a diagonal block of metric Hodge Laplacian matrices

$$\begin{aligned}  \overline{\textbf{D}}_2^2=\begin{bmatrix} \textbf{L}_{[0]} &\quad \text{0 }_{n_0\times n_1} &\quad \text{0 }_{n_0\times n_2} &\quad \text{0 }_{n_0\times n_{3}} \\ \text{0 }_{n_1\times n_0} &\quad \textbf{L}_{[1]} &\quad \text{0 }_{n_1\times n_{2}} &\quad \text{0 }_{n_1\times n_{3}} \\ \text{0 }_{n_2\times n_0} &\quad \text{0 }_{n_2\times n_1} &\quad \textbf{L}_{[2]} &\quad \text{0 }_{n_2\times n_{3}} \\ \text{0 }_{n_{3}\times n_0} &\quad \text{0 }_{n_{3}\times n_1} &\quad \text{0 }_{n_{3}\times n_2} &\quad {\textbf{L}}_{3}^{\text {down}}\\ \end{bmatrix} \end{aligned}$$

where the metric Hodge Laplacian matrices are defined as

$$\begin{aligned} \textbf{L}_{[p]} = \textbf{L}_{[p]}^{\text {down}} + \textbf{L}_{[p]}^{\text {up}}, \end{aligned}$$

with

$$\begin{aligned} \textbf{L}_{[p]}^{\text {down}}&= \textbf{B}_{p}^{\top} \textbf{G}_{p-1}^{-1}\textbf{B}_{p}\textbf{G}_{p}/(p+1),\\ \textbf{L}_{[p]}^{\text {up}}&= \textbf{G}_{p}^{-1}\textbf{B}_{p+1}\textbf{G}_{p+1}\textbf{B}_{p+1}^{\top} /(p+2). \end{aligned}$$

Depending on the matrices $\textbf{G}_{p}$, the weighted Dirac matrix may not always be symmetric, despite its eigenspectrum can be shown to be always real (see Appendix I).

For the rest of the paper, the metric matrices $\textbf{G}_{p}$ shall be defined with each metric value for a simplex $\sigma ^{p}$ to be dependent on its $(p+1)$-dimensional cofaces in the following way^63^:

$$\begin{aligned} \textbf{G}_{p}(\sigma ^{p},\sigma ^{p}) = \left\{ \begin{array}{ll} w_{\sigma ^{d}}, &\quad p=d \\ w_{\sigma ^{p}} + \displaystyle \sum _{\sigma ^{p}<\sigma ^{p+1}} \textbf{G}_{p+1}(\sigma ^{p+1}, \sigma ^{p+1}), &\quad 0\le p<d. \end{array} \right. \end{aligned}$$

Here, $w_{\sigma ^{p}}>0$ is a positive weight on *p*-simplex $\sigma ^{p}$, which can be related to physical, chemical and biological properties.

#### Discrete Dirac matrix

With the weighted Dirac matrix $\overline{\textbf{D}}_p$, a discrete Dirac matrix is simply the special case of $\overline{\textbf{D}}_p$ when $\textbf{G}_p$ are identity matrices and $a_p=1$ for all $p\ge 1$.

Previously, a general Dirac matrix has been defined as^49,69,70^

$$\begin{aligned} \textbf{D}_p(z) = \begin{bmatrix} \text{0 }_{n_p\times n_{p}} &\quad z\textbf{B}_{p+1} \\ \overline{z}\textbf{B}_{p+1}^{\top} &\quad \text{0 }_{n_{p+1}\times n_{p+1}} \end{bmatrix}, \end{aligned}$$

where $z\in {\mathbb {C}}$ such that $|z|=1$. Since $|z|=1$, the typical values of *z* occurs when $z=\overline{z}=1$ or $z=-\overline{z}=i$. In general, the parameter $z\in {\mathbb {C}}$ extends the real eigenvectors of $\textbf{D}_p(z)$ to ${\mathbb {C}}$ while the eigenvalue remains unchanged. By taking the square of the Dirac operator, we have

$$\begin{aligned} \textbf{D}_p^2 (z)= \begin{bmatrix} \textbf{L}_{p}^{\text {up}} &\quad \text{0 }_{n_p\times n_{p+1}} & \\ \text{0 }_{n_{p+1}\times n_{p}} &\quad \textbf{L}_{p+1}^{\text {down}} \end{bmatrix}, \end{aligned}$$

which implies that the eigenvalues of diagonal block real-valued Hodge-Laplacian matrices will also be the eigenvalues of $\textbf{D}_p^2 (z)$. Since the Hodge-Laplacians are positive semi-definite symmetric matrices, the eigenvalues of $\textbf{D}_p^2 (z)$ are non-negative as well. However, eigenvectors from the Dirac matrix may contain complex numbers.

For a simplicial complex ${\mathcal {K}}$ with $n_{p-1} \times n_p$ boundary matrices $\textbf{B}_p$ where $n_{p-1}$ is the number of $(p-1)$-simplices and $n_p$ is the number of *p*-simplices in ${\mathcal {K}}$, the discrete Dirac matrix $\textbf{D}_p$^70^ is

10 $$\begin{aligned} \textbf{D}_p= \begin{bmatrix} \text{0 }_{n_0\times n_0} &\quad \textbf{B}_1 &\quad \text{0 }_{n_0\times n_2} &\quad \cdots &\quad \text{0 }_{n_0\times n_{p}} &\quad \text{0 }_{n_0\times n_{p+1}} \\ \textbf{B}_1^{\top} &\quad \text{0 }_{n_1\times n_1} &\quad \textbf{B}_2 &\quad \cdots &\quad \text{0 }_{n_1\times n_{p}} &\quad \text{0 }_{n_1\times n_{p+1}} \\ \text{0 }_{n_2\times n_0} &\quad \textbf{B}_2^{\top} &\quad \text{0 }_{n_2\times n_2} &\quad \cdots &\quad \text{0 }_{n_2\times n_{p}} &\quad \text{0 }_{n_2\times n_{p+1}} \\ \vdots &\quad \vdots &\quad \vdots &\quad \vdots &\quad \vdots &\quad \vdots \\ \text{0 }_{n_{p}\times n_0} &\quad \text{0 }_{n_{p}\times n_1} &\quad \text{0 }_{n_{p}\times n_2} &\quad \cdots &\quad \text{0 }_{n_{p} \times n_{p}} &\quad \textbf{B}_{p+1}\\ \text{0 }_{n_{p+1}\times n_0} &\quad \text{0 }_{n_{p+1}\times n_1} &\quad \text{0 }_{n_{p+1}\times n_2} &\quad \cdots &\quad \textbf{B}_{p+1}^{\top} &\quad \text{0 }_{n_{p+1}\times n_{p+1}} \end{bmatrix}. \end{aligned}$$

It is of size $\sum _{i=0}^{p+1} n_i \times \sum _{i=0}^{p+1} n_i$.

**Figure 1:**
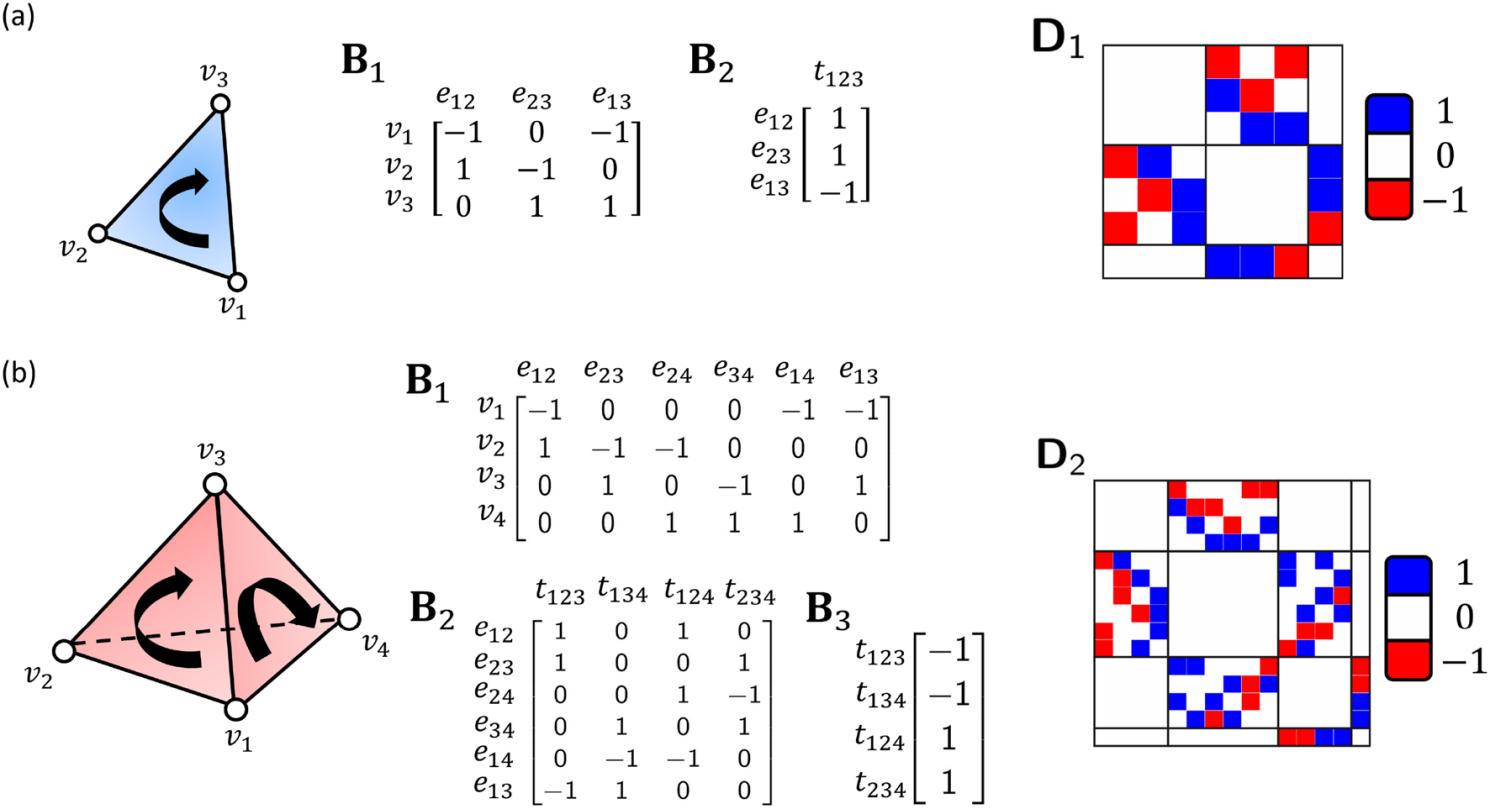
Illustration of constructions of (**a**) Discrete Dirac matrix $\textbf{D}_1$ of a triangle and (**b**) Discrete Dirac matrix $\textbf{D}_2$ of a tetrahedron along with its corresponding boundary matrices. The rows and columns of boundary matrices corresponds to a respective simplex each. For instance, in the boundary matrix $\textbf{B}_2$ of (**a**), edge $e_{12}$ is oriented similarly as $t_{123}$, hence having an entry 1 in the matrix. As the entries of Dirac operator either take a value of $-1$, 0 or 1, the entries of Dirac operators are color coded with blue indicating 1, white indicating 0 and red indicating $-1$.

Figure 1 shows a simple construction of discrete Dirac matrices (10) for a triangle and a tetrahedron. In Fig. 1a, the triangle is a 2-simplex and hence the largest Dirac operator is $\textbf{D}_1$. On the other hand, the tetrahedron in Fig. 1b is a 3-simplex and thus the largest Dirac operator is $\textbf{D}_2$.

Note that by taking the square of $\textbf{D}_p$, one would obtain a matrix with diagonal blocks of unweighted combinatorial Hodge Laplacians as shown below.

$$\begin{aligned} {\textbf{D}}_p^2=\begin{bmatrix} \textbf{L}_0 &\quad \text{0 }_{n_0\times n_1} &\quad \text{0 }_{n_0\times n_2} &\quad \cdots &\quad \text{0 }_{n_0\times n_{p}} &\quad \text{0 }_{n_0\times n_{p+1}} \\ \text{0 }_{n_1\times n_0} &\quad \textbf{L}_1 &\quad \text{0 }_{n_1\times n_{2}} &\quad \cdots &\quad \text{0 }_{n_1\times n_{p}} &\quad \text{0 }_{n_1\times n_{p+1}} \\ \text{0 }_{n_2\times n_0} &\quad \text{0 }_{n_2\times n_1} &\quad \textbf{L}_2 &\quad \cdots &\quad \text{0 }_{n_2\times n_{p}} &\quad \text{0 }_{n_2\times n_{p+1}} \\ \vdots &\quad \vdots &\quad \vdots &\quad \vdots &\quad \vdots &\quad \vdots \\ \text{0 }_{n_{p}\times n_0} &\quad \text{0 }_{n_{p}\times n_1} &\quad \text{0 }_{n_{p}\times n_2} &\quad \cdots &\quad \textbf{L}_{p} &\quad \text{0 }_{n_{p}\times n_{p+1}}\\ \text{0 }_{n_{p+1}\times n_0} &\quad \text{0 }_{n_{p+1}\times n_1} &\quad \text{0 }_{n_{p+1}\times n_2} &\quad \cdots &\quad \text{0 }_{n_{p+1}\times n_{p}} &\quad \textbf{L}_{p+1}^{\text {down}} \end{bmatrix}, \end{aligned}$$

where the unweighted Hodge Laplacian $\textbf{L}_{p}$, is given by $\textbf{L}_{p}=\textbf{L}_{p}^{\text {down}}+ \textbf{L}_{p}^{\text {up}}$ with $\textbf{L}_{p}^{\text {down}}=\textbf{B}_{p}^{\top} \textbf{B}_{p}$ and $\textbf{L}_{p}^{\text {up}}=\textbf{B}_{p+1}\textbf{B}_{p+1}^{\top}$. In our case, the last term contains only $\textbf{L}_{p+1}^{\text {down}}$.

Recall that $\textbf{B}_{p+1}^{\top} \textbf{B}_{p+1}$ is also known as the lower Hodge Laplacian $\textbf{L}_{p+1}^{\text {down}}$ while $\textbf{B}_{p+2}\textbf{B}_{p+2}^{\top}$ is known as the upper Hodge Laplacian $\textbf{L}_{p+1}^{\text {up}}$.

$$\begin{aligned} \textbf{L}_{p+1} = \textbf{L}_{p+1}^{\text {up}} + \textbf{L}_{p+1}^{\text {down}}. \end{aligned}$$

### Spectrum of the discrete Dirac operator

#### Spectral of Dirac matrix

Let $\textbf{Q}_p$ be the block diagonal matrix

$$\begin{aligned} \textbf{Q}_p=\begin{bmatrix} \textbf{I}_{n_0} &\quad \text{0 }_{n_0\times n_1} &\quad \text{0 }_{n_0\times n_2} &\quad \cdots &\quad \text{0 }_{n_0\times n_{p}} &\quad \text{0 }_{n_0\times n_{p+1}} \\ \text{0 }_{n_1\times n_0} &\quad -\textbf{I}_{n_1} &\quad \text{0 }_{n_1\times n_{2}} &\quad \cdots &\quad \text{0 }_{n_1\times n_{p}} &\quad \text{0 }_{n_1\times n_{p+1}} \\ \text{0 }_{n_2\times n_0} &\quad \text{0 }_{n_2\times n_1} &\quad \textbf{I}_{n_2} &\quad \cdots &\quad \text{0 }_{n_2\times n_{p}} &\quad \text{0 }_{n_2\times n_{p+1}} \\ \vdots &\quad \vdots &\quad \vdots &\quad \vdots &\quad \vdots &\quad \vdots \\ \text{0 }_{n_{p}\times n_0} &\quad \text{0 }_{n_{p}\times n_1} &\quad \text{0 }_{n_{p}\times n_2} &\quad \cdots &\quad (-1)^{p}\textbf{I}_{n_p} &\quad \text{0 }_{n_{p}\times n_{p+1}}\\ \text{0 }_{n_{p+1}\times n_0} &\quad \text{0 }_{n_{p+1}\times n_1} &\quad \text{0 }_{n_{p+1}\times n_2} &\quad \cdots &\quad \text{0 }_{n_{p+1}\times n_{p}} &\quad (-1)^{p+1}\textbf{I}_{n_{p+1}} \end{bmatrix}, \end{aligned}$$

where $\textbf{I}_{n_p}$ denotes an $n_p \times n_p$ identity matrix and $\textbf{Q}_p$ satisfies

$$\begin{aligned} \textbf{Q}_p^2 = \textbf{I}_{\sum _{i=0}^{p+1} n_i}. \end{aligned}$$

The Dirac matrix satisfies the supersymmetry condition $\textbf{D}_p\textbf{Q}_p = -\textbf{Q}_p\textbf{D}_p$. That also means that the anti-commutator between the Dirac matrix $\textbf{D}_p$ and block diagonal matrix $\textbf{Q}_p$ vanishes. Further,

11 $$\begin{aligned} \textbf{D}_pv=\lambda v&\iff \textbf{Q}_p\textbf{D}_p\textbf{Q}_p v = -\textbf{D}_pv = -\lambda v \nonumber \\&\iff -\textbf{Q}_p\textbf{D}_pv = -\lambda \textbf{Q}_p v\nonumber \\&\iff \textbf{D}_p\textbf{Q}_pv = -\lambda \textbf{Q}_p v, \end{aligned}$$

which implies that $\textbf{Q}_pv$ is an eigenvector associated with the eigenvalue $-\lambda$.

Essentially, the above shows that the Dirac operator of a simplicial complex satisfies $\textbf{D}_pv=\lambda v$ where $\lambda$ is the eigenvalue associated with the eigenvector *v* if and only if

$$\begin{aligned} \textbf{D}_p(\textbf{Q}_pv) = -\lambda (\textbf{Q}_pv), \end{aligned}$$

where $\textbf{Q}_pv$ is the eigenvector associated to the eigenvalue $-\lambda$.

Since $\lambda$ (resp. $-\lambda$) is an eigenvalue of $\textbf{D}_p$ with corresponding eigenvector *v* (resp. $\textbf{Q}_pv$), then for any positive integer *s*, $\lambda ^s$ (resp. $(-\lambda )^s$) is an eigenvalue of $\textbf{D}_p^s$ with corresponding eigenvector *v* (resp. $\textbf{Q}_pv$). The detailed proof is in Appendix E.

Now, we consider the relationship between the eigenspectrum of $\textbf{D}_p^2$ and $\textbf{D}_p$. For the case of zero eigenvalues, $\textbf{D}_p^2v = 0$ naturally implies $\textbf{D}_pv=0$. Hence, $\textbf{D}_p$ shares the same eigenvectors as $\textbf{D}_p^2$ for zero eigenvalues. If $\lambda ^2$ is a non-zero eigenvalue of $\textbf{D}_p^2$ with eigenvector *v*, then we have the following possible cases for $\textbf{D}_p$:

(i) $\lambda$ is an eigenvalue of $\textbf{D}_p$ with eigenvector $w=(\textbf{D}_p+\lambda I)v$. i.e. $(\textbf{D}_p-\lambda I)w=0$.
(ii) $-\lambda$ is an eigenvalue of $\textbf{D}_p$ with eigenvector $w=(\textbf{D}_p-\lambda I)v$. i.e. $(\textbf{D}_p+\lambda I)w=0$.

It is easy to derive the above cases by considering $(\textbf{D}_p^2-\lambda ^2 I)v = 0$. Then

12 $$\begin{aligned} (\textbf{D}_p-\lambda I)(\textbf{D}_p+\lambda I)v =0. \end{aligned}$$

Here, there are two possible cases since by (11), either $\lambda$ or $-\lambda$ is the eigenvalue of $\textbf{D}_p$. If $-\lambda$ is the eigenvalue of $\textbf{D}_p$, then $(\textbf{D}_p+\lambda I)w=0$ for some non-zero eigenvector *w*. This implies that $(\textbf{D}_p-\lambda I)w\ne 0$, otherwise it contradicts $(\textbf{D}_p+\lambda I)w=0$. Hence, this means that Eq. (12) can be rewritten as

$$\begin{aligned} (\textbf{D}_p+\lambda I)w=0, \end{aligned}$$

where $w=(\textbf{D}_p-\lambda I)v$ is a non-zero eigenvector for $\textbf{D}_p$ with corresponding eigenvalue $-\lambda$.

Similarly, if $\lambda$ is an eigenvalue of $\textbf{D}_p$, then $(\textbf{D}_p-\lambda I)w=0$ for some non-zero eigenvector *w*. This implies that $(\textbf{D}_p+\lambda I)w\ne 0$, otherwise it contradicts $(\textbf{D}_p-\lambda I)w=0$. Therefore, Eq. (12) can be rewritten as

$$\begin{aligned} (\textbf{D}_p-\lambda I)w=0, \end{aligned}$$

where $w=(\textbf{D}_p+\lambda I)v$ is a non-zero eigenvector for $\textbf{D}_p$ with corresponding eigenvalue $\lambda$.

This leads us to the following relations connecting $\textbf{D}_p$, $\textbf{D}_p^2$ and $\textbf{L}_k$ ($0\le k \le p+1$). For any $v\in \ker \textbf{D}_p^2$,

$$\begin{aligned} \textbf{D}_p^2 v = \text{0 }&\iff {\left\{ \begin{array}{ll} \textbf{L}_0\textbf{w}_0 = \text{0 }, &\quad k=0\\ \textbf{L}_k\textbf{w}_k = \text{0 }, &\quad 0<k<p+1\\ \textbf{L}_{p+1}^{\text {down}}\textbf{w}_{p+1} = \text{0 }, &\quad k = p+1 \end{array}\right. }, \end{aligned}$$

where $v = (\textbf{w}_0^{\top} , \textbf{w}_1^{\top} ,\ldots ,\textbf{w}_{k-1}^{\top} ,\textbf{w}_k^{\top} ,\textbf{w}_{k+1}^{\top} ,\ldots ,\textbf{w}_p^{\top} ,\textbf{w}_{p+1}^{\top} )^{\top}$. In other words, *v* is a vector consisting of block vectors $\textbf{w}_k^{\top}$ for $0\le k\le p+1$. This means that for every $0 \le k \le p$, $\textbf{w}_k^{\top} \in \ker \textbf{L}_k$. In the case where $k=p+1$, $\textbf{w}_{p+1}^{\top} \in \ker \textbf{L}_{p+1}^{\text {down}}$. We have,

$$\begin{aligned} (\textbf{w}_0^{\top} , \textbf{w}_1^{\top} ,\ldots ,\textbf{w}_{p+1}^{\top} )^{\top} \in \ker \textbf{L}_{p+1}^{\text {down}} \oplus \bigoplus _{k=0}^{p} \ker \textbf{L}_k. \end{aligned}$$

Note that for $\textbf{w}_{p+1}^{\top}$, it is the eigenvector from the kernel of $\textbf{L}_{p+1}^{\text {down}}$.

Hence, the kernel of $\textbf{D}_p^2$ can be decomposed into a direct sum of kernels of $\textbf{L}_k$ from $k=0$ to $k=p+1$:

$$\begin{aligned} \ker \textbf{D}_p^2 = \ker \textbf{L}_{p+1}^{\text {down}} \oplus \bigoplus _{k=0}^{p} \ker \textbf{L}_k. \end{aligned}$$

Further, we have

13 $$\begin{aligned} \ker \textbf{D}_p = \ker \textbf{D}_p^2&= \ker \textbf{L}_{p+1}^{\text {down}} \oplus \bigoplus _{k=0}^{p} \ker \textbf{L}_k \nonumber \\&\cong \ker \textbf{L}_{p+1}^{\text {down}} \oplus \bigoplus _{k=0}^{p} H_k, \end{aligned}$$

where $\bigoplus _{k=0}^{p} H_k$ refers to the direct sum of homology groups.

Therefore, the eigenvectors of $\textbf{D}_p$ reveal both *k*-th homology and *k*-th non-homology information within the structural data for all $0\le k \le p+1$. Instead of eigendecomposing HL matrices for all $0\le k \le p+1$, one can simply eigendecompose $\textbf{D}_p$ to obtain all of the eigenspectrums. As the number of zero eigenvalues of $\textbf{L}_{p+1}^{\text {down}}$ is the $\text {rank }\textbf{B}_{p+2}^{\top}$ plus the $(p+1)$-th Betti number $\beta _{p+1}$, the multiplicity of zero eigenvalues in $\textbf{D}_p$ is the $\text {rank }\textbf{B}_{p+2}^{\top}$ plus the total sum of all the Betti numbers from dimension 0 to $p+1$. That is,

14 $$\dim \ker \textbf{D}_p = \text {rank } \textbf{B}_{p+2}^{\top} + \sum _{k=0}^{p+1}\beta _k.$$

**Figure 2:**
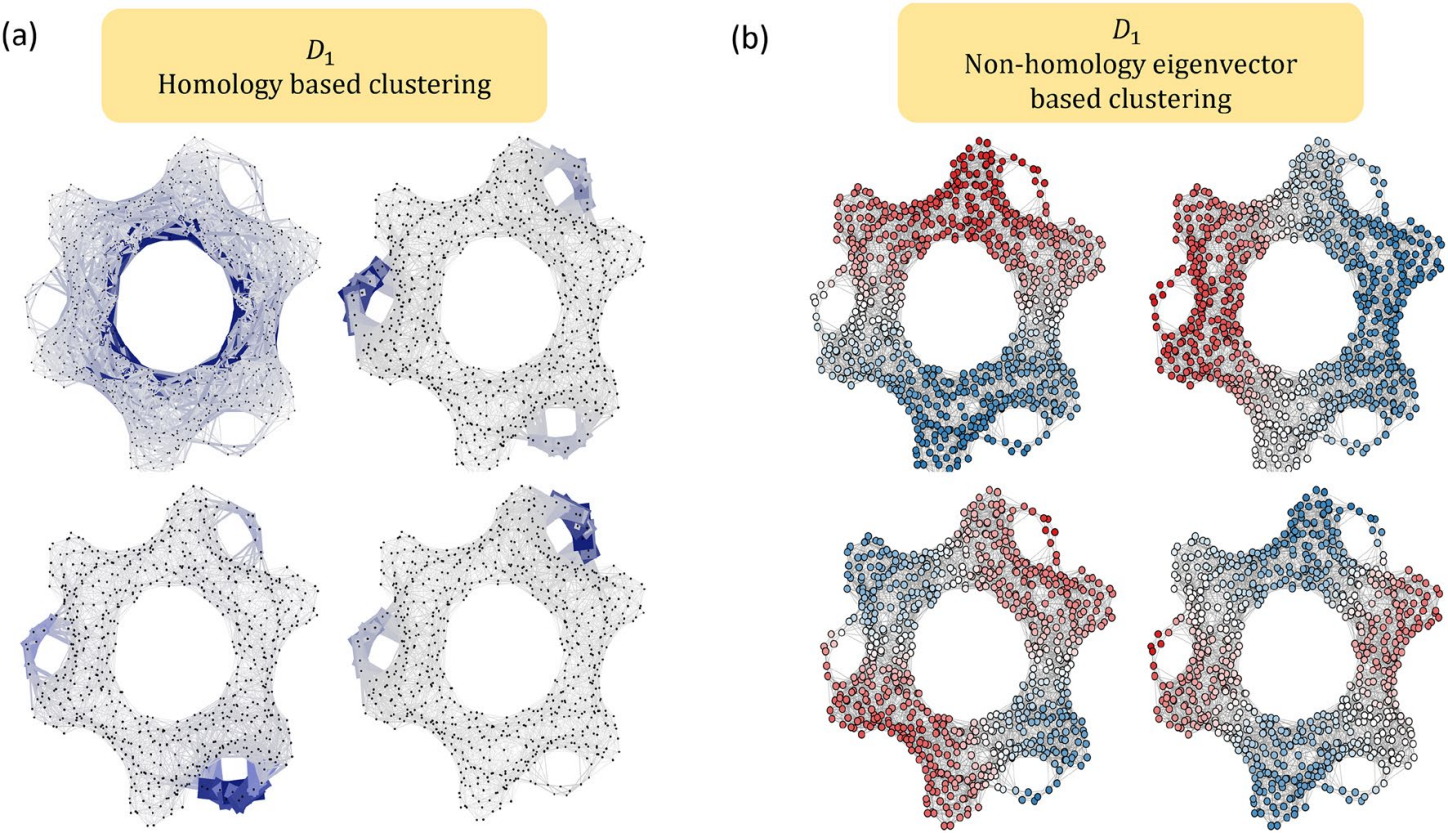
Illustration of loop/circle-based clustering using four one-dimensional (1D) homology generators (**a**) and spectral clustering using four zero-dimensional (0D) non-homology generators (**b**). The Dirac matrices $\textbf{D}_1$ are generated from the Vietoris Rips complex of the C$_\alpha$ atoms in PDBID: 1AXC at 10Å. (**a**) Here 1D homology generators $\textbf{w}_1^{\top}$ are taken from the homology generators of $\textbf{D}_1$ with eigenvalues as 0. A thick edge with dark blue color indicates large magnitude of the value, while a thinner edge with light blue color means the corresponding the 1D homology generator has a value with small magnitude on this 1-simplex. Each 1D homology generator forms an individual loop or circle. (**b**) The four 0D non-homology generators $\textbf{w}_0^{\top}$ are taken from the non-homology generators of $\textbf{D}_1$ with the four smallest positive eigenvalues. Note that these 0D non-homology generators are defined on nodes (0-simplices). Nodes with negative values are colored in red while nodes with positive values are of blue color. It can be seen that the nodes in the structure can be naturally clustered into groups based on the signs of these 0D non-homology generators.

Mathematically, the eigenvectors corresponding to the zero eigenvalues are known as homology generators while those from non-zero eigenvalues are the non-homology generators. Both of them can be used in structural clustering. More specifically, the homology generators can be used for clustering structures based on their loop or circle components, while non-homology generators are related to the spectral clustering, in which communities and clusters are based on their distances. Figure 2 demonstrates the structural clustering with homology and non-homology generators for a protein (PDBID: 1AXC). We only consider the C$_\alpha$ atoms in structure. A Vietoris Rips complex is constructed by using a cutoff distance of 10Å. The Dirac matrix $\textbf{D}_1$ and its eigenvalues and eigenvectors are calculated. As the non-zero eigenvalues of $\textbf{D}_1$ come in pairs, it suffices to consider the eigenvectors corresponding to the positive eigenvalues. For all the non-negative eigenvalues of $\textbf{D}_1$, the eigenvectors are arranged in ascending order according to its corresponding eigenvalues.

Figure 2a illustrates the loop/circle-based clustering using four one-dimensional (1D) homology generators. Note that these 1D homology generators $\textbf{w}_1^{\top}$ are taken from the homology generators of $\textbf{D}_1$ (with eigenvalues 0). More specifically, these 1D homology generators are defined by the 1-simplices. In Fig. 2a, a thick edge with dark blue color indicates large magnitude of the value, while a thinner edge with light blue color means the corresponding 1D homology generator has a value with small magnitude on this 1-simplex. It can be seen that edges with large magnitudes are the 1-simplices that form circles or loops. Each 1D homology generator forms an individual loop or circle. In this way, 1D homology generators can be used for loop/circle-based clustering of molecular structures.

Figure 2b illustrates the spectral clustering using four zero-dimensional (0D) non-homology generators. The four 0D non-homology generators $\textbf{w}_0^{\top}$ are taken from the non-homology generators of $\textbf{D}_1$ with the four smallest positive eigenvalues. Note that these 0D non-homology generators are defined on nodes (0-simplices). In Fig. 2b, nodes with negative values are colored in red while nodes with positive values are of blue color. It can be seen that the nodes in the structure can be naturally clustered into groups based on the signs of these 0D non-homology generators. This approach is known as spectral clustering and widely used in data analysis. It should be noticed that using the higher order Dirac matrices, we can cluster not only nodes (0-simplices), but also higher dimensional simplices.

**Figure 3:**
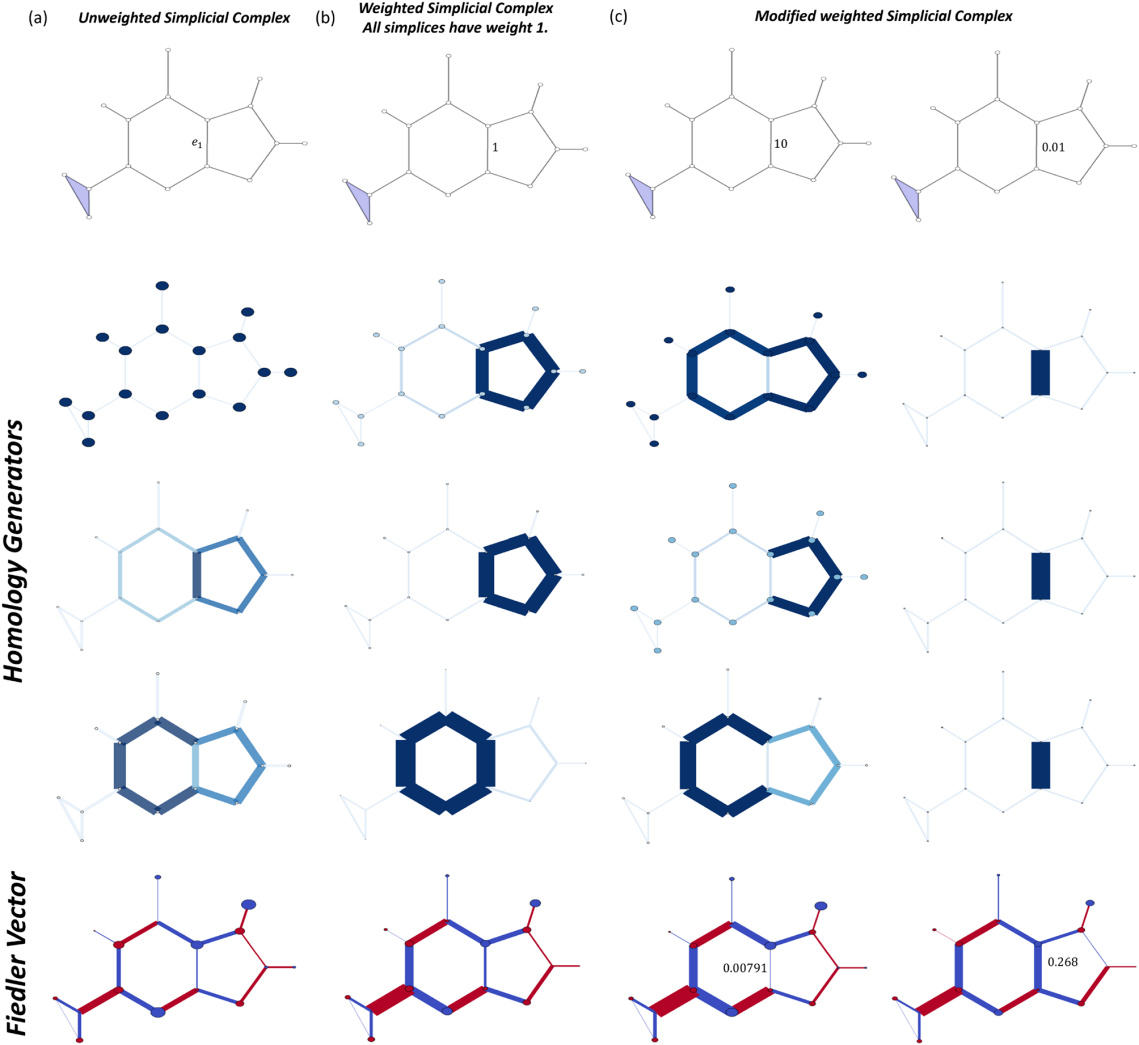
Illustration of three homology generators and Fiedler vector from (**a**) discrete Dirac matrix and (**b**,**c**) weighted Dirac matrix (from weighted simplicial complexes). For the discrete Dirac matrix, the three homology generators represents one 1D component and two 2D circles. By assigning simplex $\sigma$ with different weight $w_\sigma$s, three weighted simplicial complexes are constructed in (**b**) and (**c**). In (**b**), the weighted simplicial complex consists of all weights $w_\sigma$ equal to 1. (**c**) Shows two weighted simplicial complexes by changing the weights of edge $e_1$ from 1 to 10 and 0.01 while the rest of weights remain unchanged. The magnitude of the homology generators are influenced by these weights and are reflected based on their thickness and darkness. For the homology generators, the edges (or vertices) are thicker and in darker blue color if they have a larger magnitude. Similarly, the edges and vertices are colored in red/blue if their elements in the Fiedler vectors have positive/negative sign. The magnitudes of their values in Fiedler vectors are represented by the thickness of edges and size of vertices.

#### Spectral of weighted Dirac matrix

The weighted Dirac matrix has different spectral properties based on the different weighting schemes. Figure 3 illustrates the spectrum of the weighted Dirac matrix defined from the guanine molecule structure (using all-atom representation). We construct an unweighted Vietoris Rips complex using a cutoff distance of 1.2Å. The discrete Dirac matrix $\textbf{D}_1$ can be computed using (10). The discrete Dirac matrix $\textbf{D}_1$ is eigendecomposed to obtain its eigenvalues and eigenvectors. Moreover, a weighted simplicial complex is constructed by assigning simplex $\sigma$ with different weight $w_\sigma$. The metric matrices $\textbf{G}_p$ are computed and weighted Dirac matrix $\overline{\textbf{D}}_1$ can then be constructed. Figure 3 shows the homology generators and Fiedler vector for an unweighted simplicial complex and three different weighted simplicial complexes. Among the three weighted simplicial complexes, Fig. 3b shows a weighted simplicial complex where all weights $w_\sigma$ are equal to 1. Two modified weighted simplicial complexes are constructed by modifying the weights of edge $e_1$ ranging from 10 and 0.01 with all the other weights kept unchanged. With the same underlying simplicial complex, they share the same three homology generators, one 1D component and two 2D circles. Figure 3 shows the corresponding eigenvectors for these homology generators. The magnitude of the eigenvectors are represented by the thickness and darkness. An edge (or vertex) with thicker lines and darker blue color indicates a larger magnitude.

In general, the weight of a simplex has an inverse effect on the corresponding element of the homology eigenvectors (i.e., homology generators). When the simplex has a smaller weight, the corresponding element of the homology eigenvectors has larger magnitude. Similar patterns also appear in non-homology generators. Fig. 3 illustrates the Fiedler vectors (i.e., eigenvector corresponding to the first smallest non-zero eigenvalue) of the nonweighted and weighted simplicial complexes. Simplices are colored in red/blue if the element of the non-homology eigenvectors has value positive/negative. The thickness of simplices represents the magnitude of their values in non-homology generators. It can be seen clearly that the weight of a simplex has an inverse effect on its magnitude of the values of eigenvectors.

## Results

### Persistent Dirac

#### Mathematical foundation for persistent Dirac analysis

Recently, persistent Laplacian and persistent sheaf Laplacians have been developed^61,62^. Their essential idea is to explore the persistence of spectral information during the filtration process. Here we develop the rigorous mathematical framework for persistent Dirac.

Let $({\mathbb {R}}, \le )$ be a category of real numbers with morphisms given by $a\rightarrow b$ for any $a\le b$. A functor $\mathcal {F}:({\mathbb {R}}, \le ) \rightarrow {\textbf {Simp}}$ gives a filtration of simplicial complexes of finite type, i.e. $\mathcal {F}$ maps from a category of real numbers to a category of simplicial complexes of finite type. For any two real numbers $a\le b$, the functor $\mathcal {F}$ satisfies the inclusion

$$\begin{aligned} \mathcal {F}(a) \hookrightarrow \mathcal {F}(b), \end{aligned}$$

which induces a morphism of chain complexes

$$\begin{aligned} C_*(\mathcal {F}(a), {\mathbb {R}}) \hookrightarrow C_*(\mathcal {F}(b), {\mathbb {R}}). \end{aligned}$$

Let $\mathcal {F}(\infty )=\bigcup _{a\in {\mathbb {R}}} \mathcal {F}(a)$ and $C_*=C_*(\mathcal {F}(\infty ), {\mathbb {R}})$. Note that $C_*$ can be endowed with an innerproduct $\langle \cdot ,\cdot \rangle$. Further, a subspace $C_*(\mathcal {F}(a), {\mathbb {R}})$ would inherit the inner product structure of $C_*$ and a boundary operator given by the restriction

$$\begin{aligned} \partial ^a_p = \partial _p|_{C_p(\mathcal {F}(a),{\mathbb {R}})}: C_p(\mathcal {F}(a), {\mathbb {R}}) \rightarrow C_{p-1}(\mathcal {F}(a), {\mathbb {R}}). \end{aligned}$$

Here $\partial _*$ is the boundary operator of $C_*$. For convenience, we shall write $C^a_p = C_p(\mathcal {F}(a), {\mathbb {R}})$. For a pair of simplicial complexes $\mathcal {F}(a) \subset \mathcal {F}(b)$, we consider the inclusion map $\iota :\mathcal {F}(a)\hookrightarrow \mathcal {F}(b)$. For $p\in \mathbb N$, the subspace

$$\begin{aligned} C_p^{a,b}:= \{x\in C^b_p: \partial _p^b(x)\in C^a_{p-1}\} \subseteq C^b_p, \end{aligned}$$

which consists of the *p*-chains in $C^b_p$ such that their images are under the boundary operator $\partial _p^b$ in the subspace $C^a_{p-1}$ of $C^b_{p-1}$. Also, we have a linear operator

$$\begin{aligned} \partial _{p}^{a,b} = \partial _p^b|_{C_p^{a,b}}: C_p^{a,b} \rightarrow C_{p-1}^{a}, \end{aligned}$$

which induces an adjoint operator

$$\begin{aligned} (\partial _{p}^{a,b})^*: C_{p-1}^a \rightarrow C_p^{a,b} \end{aligned}$$

with respect to the inner product $\langle \cdot ,\cdot \rangle$.

Let $n^{a,b}_p:= \dim (C^{a,b}_p)$. Then following commutative diagram is thus induced by $\iota$.
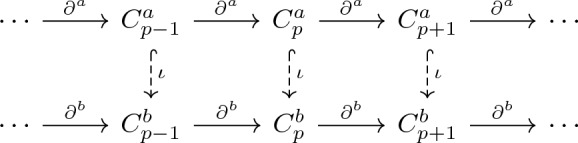


Notice that $\partial _p^{a,b}$ is a restriction to $C_p^{a,b}$ in order to obtain the “diagonal” operators $\partial _p^{a,b} : C_p^{a,b} \rightarrow C_{p-1}^a$. Similarly, with a restriction to $C_p^{a,b}$, we can then define the *p*-dimensional boundary matrices $\textbf{B}_{p}^{a,b}$ which consists of every entry value of $\partial _p^{a,b}$.

The persistent Dirac operator $\textbf{D}_{p}^{a,b}$ can then be written as follows.

$$\begin{aligned} \textbf{D}_{p}^{a,b}=\begin{bmatrix} \text{0 }_{n_0\times n_0} & \textbf{B}_{1}^{a,b} & \text{0 }_{n_0\times n_2} & \cdots & \text{0 }_{n_0\times n_{p}} & \text{0 }_{n_0\times n_{p+1}} \\ (\textbf{B}_{1}^{a,b})^{\top} & \text{0 }_{n_1\times n_1} & \textbf{B}_{2}^{a,b} & \cdots & \text{0 }_{n_1\times n_{p}} & \text{0 }_{n_1\times n_{p+1}} \\ \text{0 }_{n_2\times n_0} & (\textbf{B}_{2}^{a,b})^{\top} & \text{0 }_{n_2\times n_2} & \cdots & \text{0 }_{n_2\times n_{p}} & \text{0 }_{n_2\times n_{p+1}} \\ \vdots & \vdots & \vdots & \vdots & \vdots & \vdots \\ \text{0 }_{n_{p}\times n_0} & \text{0 }_{n_{p}\times n_1} & \text{0 }_{n_{p}\times n_2} & \cdots & \text{0 }_{n_{p} \times n_{p}} & \textbf{B}_{p}^{a,b}\\ \text{0 }_{n_{p+1}\times n_0} & \text{0 }_{n_{p+1}\times n_1} & \text{0 }_{n_{p+1}\times n_2} & \cdots & (\textbf{B}_{p}^{a,b})^{\top} & \text{0 }_{n_{p+1}\times n_{p+1}} \end{bmatrix}. \end{aligned}$$

The maps and spaces are also illustrated in the diagram below
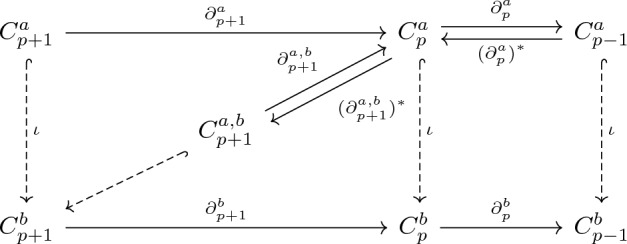


Further, the *p*-th persistent Hodge Laplacian can be defined as

$$\begin{aligned} \textbf{L}_p^{a,b} = {\left\{ \begin{array}{ll} \textbf{B}_{1}^{a,b}(\textbf{B}_{1}^{a,b})^{\top} , &\quad p =0 \\ (\textbf{B}_p^{a,b})^{\top} \textbf{B}_p^{a,b} + \textbf{B}_{p+1}^{a,b}(\textbf{B}_{p+1}^{a,b})^{\top} , &\quad p>0. \end{array}\right. } \end{aligned}$$

Similarly, the matrices $(\textbf{B}_p^{a,b})^{\top} \textbf{B}_p^{a,b}$ and $\textbf{B}_{p+1}^{a,b}(\textbf{B}_{p+1}^{a,b})^{\top}$ are the *p*-th persistent lower and upper Hodge Laplacians $(\textbf{L}_{p+1}^{\text {down}})^{a,b}$ and $(\textbf{L}_{p+1}^{\text {up}})^{a,b}$ respectively. Based on (13), the following result shows that the nullity of *p*-th persistent Dirac operator equals to the rank of $\textbf{B}_{p+2}^{\top}$ plus the sum of *k*-th persistent Betti numbers, where $0\le k \le p+1$.

$$\begin{aligned} \ker \textbf{D}_p^{a,b} = \ker (\textbf{D}_p^{a,b})^2&= \ker (\textbf{L}_{p+1}^{\text {down}})^{a,b} \oplus \bigoplus _{k=0}^{p} \ker \textbf{L}_k^{a,b} \\ &\cong \ker (\textbf{L}_{p+1}^{\text {down}})^{a,b} \oplus \bigoplus _{k=0}^{p} (H_k)^{a,b}, \end{aligned}$$

where $\bigoplus _{k=0}^{p} (H_k)^{a,b}$ refers to the direct sum of (*a*, *b*)-persistent homology groups. The (*a*, *b*)-persistent homology groups characterizes the homology generators that are born at time *a* and survive to time *b*.

**Figure 4:**
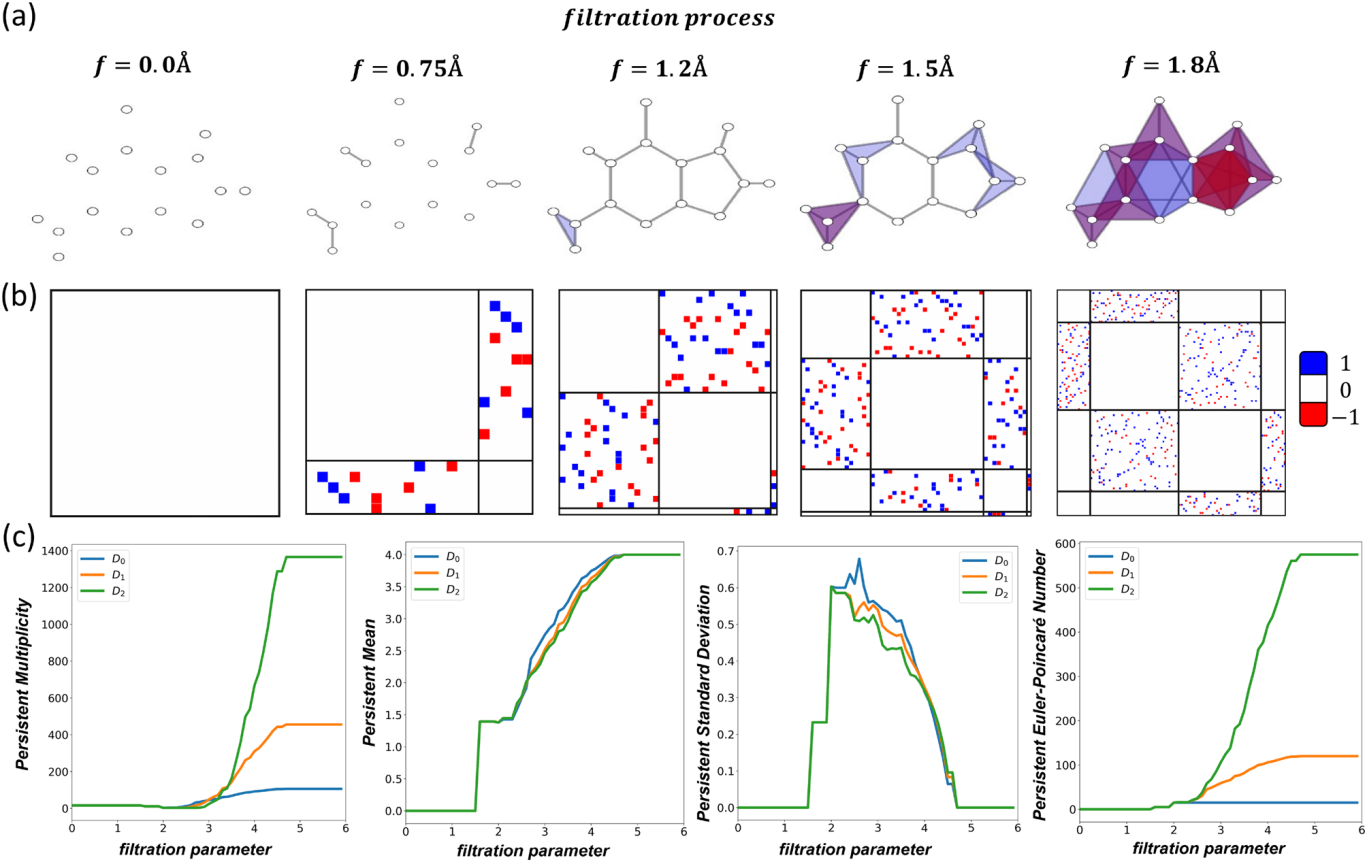
Illustration of the filtration process of the guanine molecule (**a**), its associated Dirac matrices (**b**), and persistent attributes (**c**). In the filtration process, more simplices are formed in simplicial complex and the size of Dirac matrix increases. The eigenspectrum of Dirac matrices changes in the filtration process. The changes in eigenspectrum are being converted into a series of 12 statistical and combinatorial attributes (i–xii). One of the statistical attribute, persistent multiplicity, provides quantitative analysis to the change in zero eigenvalues of Dirac matrices while the remaining 11 persistent attributes are derived from the non-zero eigenvalues.

Figure 4 illustrates the persistent Dirac analysis of the guanine molecule (using all-atom representation). More specifically, Fig. 4a shows the Vietoris–Rips complex of the guanine molecule when filtration parameter $f= 0.0$Å, 0.75Å, 1.2Å, 1.5Å and 1.8Å. In particular, triangles first appear around 1.2Å and tetrahedron starts to appear at 1.5Å. Figure 4b shows the corresponding Dirac matrix $\textbf{D}_2$. The size of the Dirac matrix $\textbf{D}_2$ consistently increases during the filtration process.

#### Persistent attributes

For any Dirac matrix, its non-zero eigenvalues come in pairs. Each pair contains one negative eigenvalue and one positive counterpart. For the set of all its positive eigenvalues, a Dirac Zeta function can be defined as follows^69^,

$$\begin{aligned} \zeta (s) = \sum _{j=1}^n \frac{1}{\lambda _j^s} = \sum _{j=1}^n e^{-s\log \lambda _j}, s\in \mathbb {C}. \end{aligned}$$

Here $\zeta (-m) = \sum _{i=1}^n \lambda _i^m$, $m\in \mathbb {Z}$ is the *m*-th spectral moments of Dirac matrices and $\zeta (-1)$ is the Laplacian graph energy. Another way to define Dirac Zeta function is to consider its negative eigenvalues by replacing the $\lambda _j^{-s}$ with $(1+e^{-i\pi s})|\lambda _j|^{-s}$. Here $\lambda _j$ can be negative. For instance, $\zeta (2) = 2\sum _{j=1} \lambda _j^{-2}$.

Furthermore, the *q*-Dirac complexity of a simplicial complex ${\mathcal {K}}$ can be defined as

$$\begin{aligned} c_q(\textbf{D}_p) = \prod _{\begin{array}{c} \lambda _j\ne 0\\ \lambda _j\in \sigma (\textbf{D}_p) \end{array}} \lambda _j^q. \end{aligned}$$

The case where $q=1$ is previously introduced by Knill^70^. $c_1(\textbf{D}_p)$ is equal to the product of all non-zero eigenvalues in spectra of $\textbf{D}_p$ since the non-zero eigenvalues come in pairs. The number of non-zero eigenvalues pairs in $\textbf{D}_p$ is the (signless) Euler–Poincaré number defined as follows,

$$\begin{aligned} \ell = \frac{1}{2}\sum _{k=0}^{p+1} n_k-\frac{1}{2}\dim \ker \textbf{D}_p \end{aligned}$$

where $n_k$ is the number of *k*-simplices and $\dim \ker \textbf{D}_p$ is the multiplicity of zero eigenvalues of $\textbf{D}_p$.

Using Eq. (14), $\ell$ can be computed as follows:

15 $$\begin{aligned} \ell = \frac{1}{2}\sum _{k=0}^{p+1} (n_k - \beta _k) - \frac{1}{2}\text {rank } \textbf{B}_{p+2}^{\top} . \end{aligned}$$

Interestingly, the spanning tree number, introduced as one of the spectral indices in molecular descriptors^1^, can be written as

$$\begin{aligned} t(\textbf{D}_p) = \frac{1}{2}\log (c_1(\textbf{D}_p)) - \log (\ell +1), \end{aligned}$$

Alternatively, $$t(\textbf{D}_p) = \log \left[ \frac{1}{\ell +1}\cdot \sqrt{c_1(\textbf{D}_p)}\right]$$.

To summarize, we consolidate and consider 11 statistical and combinatorial attributes as molecular descriptors for each given set of positive eigenvalues $\{\lambda _1, \lambda _2, \ldots , \lambda _\ell \}$ where $\ell$ is the number of non-zero eigenvalue pairs:

(i) $\min \{\lambda _1, \lambda _2, \ldots , \lambda _k\}$, also known as the Fiedler value.
(ii) $$\max \{\lambda _1, \lambda _2, \ldots , \lambda _n\}$$
(iii) $$\bar{\lambda } = \frac{1}{n}\sum _{i=1}^n \lambda _i = \frac{1}{n}\zeta (-1)$$.
(iv) Standard Deviation
(v) Laplacian Graph Energy $\zeta (-1)$.
(vi) (Signless) Euler–Poincaré Number (number of non-zero eigenvalue pairs) $\ell$
(vii) Generalised Mean Graph Energy $\sum _{i=1}^n \frac{|\lambda _i-\bar{\lambda }|}{n}$.
(viii) Spectral 2nd Moment $\zeta (-2)$.
(ix) $$\zeta (2) = 2\sum _{j=1}^n \lambda _j^{-2}$$.
(x) Quasi-Wiener Index $(n+1)\zeta (1)$.
(xi) Spanning Tree Number $t(\textbf{D}_p)$.

In addition to the 11 statistical attributes, we also consider the persistent multiplicity of zero eigenvalues.

(xii) Persistent Multiplicity of zero eigenvalues.

Figure 4c shows the persistent multiplicity, persistent mean, persistent standard deviation and persistent (signless) Euler–Poincaré number for the filtration of guanine molecule. Further information such as the persistent multiplicities of $\textbf{L}_k$ ($0\le k\le 2)$ and $\textbf{L}_k^{\text {down}}$ ($1\le k \le 3$) can be found in Appendix G. Recall that the persistent multiplicity is equivalent to the persistent Betti number. Here, the persistent multiplicity and persistent (signless) Euler–Poincaré number of $\textbf{D}_p$ can be quantitatively analysed by comparing the persistent multiplicity of $\textbf{L}_{p+1}^{\text {down}}$ and the *k*-th persistent Betti numbers for $0\le k \le p$. It can be seen that these persistent attributes change with the filtration value. Each variation of the persistent attribute indicates a certain change in the simplicial complex.

At the very start of the filtration, there are 16 isolated atoms which means that there are 16 connected components. Hence, the persistent multiplicity of $\textbf{L}_0$ is 16 since $\beta _0 = 16$. As all other Betti numbers are zero and there are no higher order simplices present at the start of the filtration, $\textbf{D}_0$, $\textbf{D}_1$ and $\textbf{D}_2$ are all-zero $16\times 16$ matrices. Therefore, the persistent multiplicity of $\textbf{D}_0$, $\textbf{D}_1$ and $\textbf{D}_2$ are all equal to 16. Using Eq. (15), the persistent (signless) Euler–Poincaré number is zero.

As filtration parameter *f* increases, the size of $\textbf{D}_0$, $\textbf{D}_1$ and $\textbf{D}_2$ matrix increases as well. This differs from the Hodge Laplacian matrix $\textbf{L}_0$, whose size remains unchanged.

At filtration size 4.7Å, a complete simplicial complex is achieved, i.e., any $p+1$ vertices will form a *p*-simplex. When this happens, the size of $\textbf{D}_p$ no longer increases any further. Here, the size of $\textbf{D}_0$, $\textbf{D}_1$ and $\textbf{D}_2$ are distinct. The size of $\textbf{D}_0$ is $136\times 136$ since $\frac{16\times 15}{2}$ (no. of 1-simplices) + 16 (no. of 0-simplices) = 136. Similarly, the sizes of $\textbf{D}_1$ and $\textbf{D}_2$ are $696\times 696$ and $2516\times 2516$ respectively. Furthermore, the persistent multiplicity of $\textbf{D}_0$, $\textbf{D}_1$ and $\textbf{D}_2$ are also distinct. Using Eq. (14), the persistent multiplicity of $\textbf{D}_0$ is 105 (persistent multiplicity of $\textbf{L}_{1}^{\text {down}}$) and 1 (0-dimensional persistent Betti number) which sums up to 106. Since the persistent multiplicity of $\textbf{L}_1$ (see Appendix G) is zero, then Eq. (3) implies that the rank of $\textbf{B}_2^{\top}$ is 105. In addition, the persistent multiplicity of $\textbf{D}_1$ and $\textbf{D}_2$ are 456 and 1366 respectively. Based on the non-zero eigenvalues, the persistent (signless) Euler–Poincaré number of $\textbf{D}_0$, $\textbf{D}_1$ and $\textbf{D}_2$ is 15, 120 and 575 due to Eq. (15).

### Persistent Dirac for molecular structure representation

Recently, a series of persistent models, including persistent homology, persistent spectral, persistent Ricci curvature, and persistent Laplacian, have demonstrated their great power in molecular representations^3,6,38,71^. They have consistently outperformed traditional graph-based models in various tasks of drug design. Here we study the representation capability of Persistent Dirac in molecular data analysis.

We consider the organic-inorganic halide perovskite (OIHP) dataset. More specifically, three kinds of Methylammonium lead halides (MAPbX$_3$, X$=$Cl, Br, I), i.e., orthorhombic, tetragonal, and cubic phase of MAPbX$_3$ are used. For each kind, there are 3 types of X atoms, including chlorine Cl, bromine Br and iodine I. The molecular dynamic simulations are systematically carried out on these molecular structures with the initial configurations based on pre-defined crystal cell parameters. For each MAPbX$_3$ structure, 1000 configurations are equally sampled from its MD simulation trajectory and the last 500 configurations, which represent stable structures, are selected for the test of our persistent Dirac model. Essentially, a total of 4500 configurations from the 9 types of MAPbX$_3$ structures are mixed together and our persistent Dirac based molecular fingerprint is used in the clustering of these configurations.

Computationally, our persistent Dirac is generated based on Alpha complex and the filtration parameter is the circumradius. More specifically, for each frame, an Alpha complex is constructed based on Delauney triangulation and circumradius of the simplex. The Dirac matrices $\textbf{D}_0$ and $\textbf{D}_1$ are computed from 1Å to 6.5Å with stepsize 0.25Å throughout the filtration process. Hence, the eigenvalues of $\textbf{D}_0$ and $\textbf{D}_1$ each contribute to 12 statistical attributes (i–xii) for 23 timesteps per frame. The feature size sums up to 552. By considering with and without hydrogen atoms, the total feature size for persistent Dirac is $552\times 2 = 1104$. Likewise, for coordinate-only model, the input features are *xyz*-coordinates of all the atoms. Since each structure consists of 553 atoms, the feature size is of $553\times 3=1659$. For the discrete Dirac model, the feature size is 552. The clustering of these MAPbX$_3$ structures is then studied using unsupervised learning models, in particular *t*-distributed stochastic neighbor embedding (*t*-SNE).

**Figure 5:**
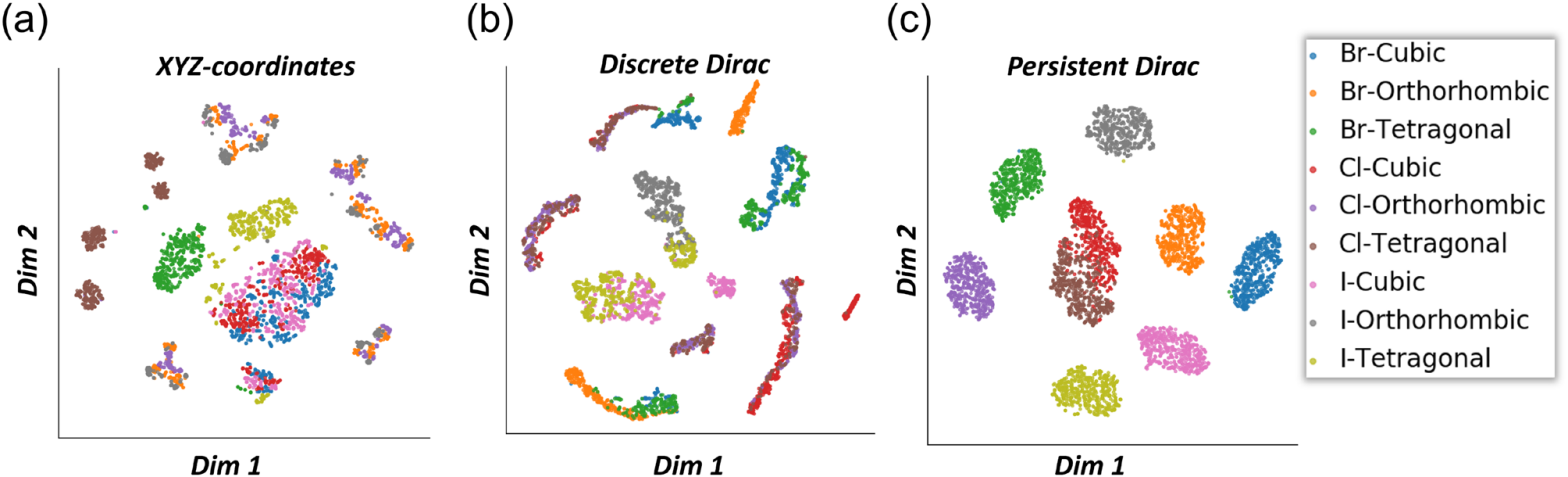
The clustering of 9 types of OIHP molecular dynamics (MD) trajectories. Three feature generation schemes are considered, including (**a**) $XYZ$-coordinates, (**b**) Discrete Dirac at 3.5Å and (**c**) Persistent Dirac. Each trajectory contains 1000 configurations and *t*-SNE model is used for clustering (of the last 500 configurations at equilibrium). The x-axis and y-axis are the two principal components obtained from the *t*-SNE model.

Figure 5 illustrates the comparison of the clustering results from three different models, including coordinate-only model ($XYZ$-coordinate) (a), discrete Dirac (b), and persistent Dirac (c). It can be seen that our persistent Dirac model demonstrates better capabilities in characterizing the intrinsic structure information and discriminating the 9 types of OIHPs clearly. In our persistent Dirac model, the filtration process at various scales provided the geometrical information needed to balance the topological information. The combination of topological and geometrical information contributes to the success of our persistent Dirac model in OIHP clustering. Figure 5b shows the performance of Dirac matrix related statistical attributes at filtration value 3.5Å. Even though it shows certain clustering effects, the overall performance is not as good as persistent Dirac. Additional clustering tests are performed for discrete Dirac model at 3Å and 4Å in Appendix F. Similarly, statistical attributes of discrete Dirac model at a single scale fail to distinguish the 9 types of OIHPs.

## Persistent Dirac for solvation free energy prediction

**Figure 6:**
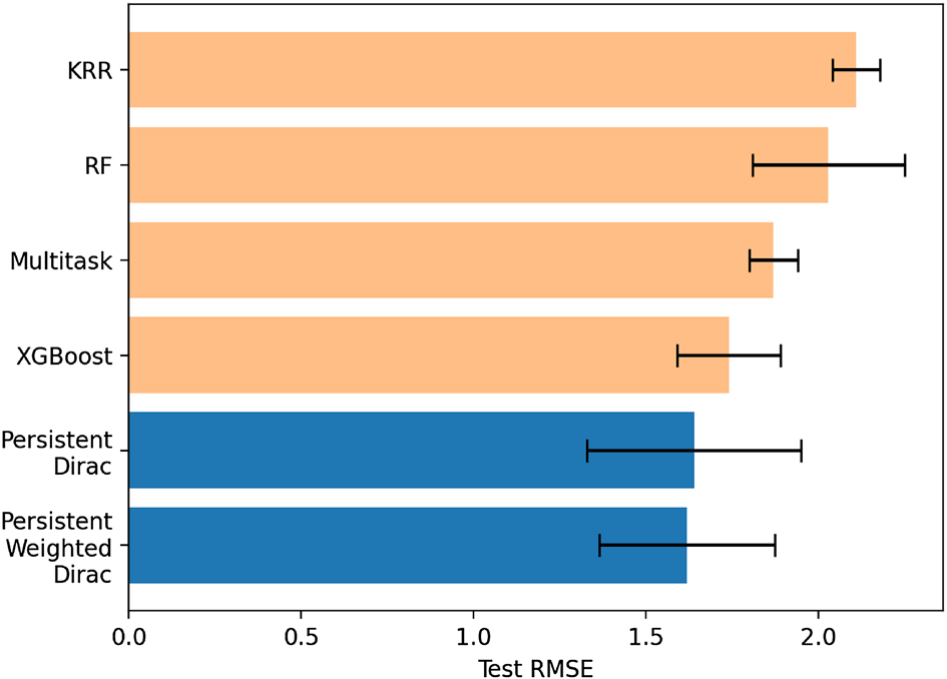
The comparison of persistent Dirac and persistent weighted Dirac models with conventional methods from MoleculeNet^74^ on the FreeSolv database. Both types of persistent Dirac models performed better than existing conventional methods such as MoleculeNet’s XGBoost (1.74 ± 0.15 kcal/mol), Multitask (1.87 ± 0.07 kcal/mol), Random Forest (RF) (2.03 ± 0.22 kcal/mol) and Kernel Ridge Regression (KRR) (2.11 ± 0.07 kcal/mol) models. Note that a lower RMSE value indicates better result.

In order to further validate the capabilities of persistent Dirac models, we perform a preliminary test of our persistent Dirac model on the Free Solvation (FreeSolv) database^72^. The FreeSolv database contains 643 SMILES sequences for small molecules and their solvation free energy values in water^73^. The FreeSolv database is also one of the physical chemistry benchmark tasks in MoleculeNet^74^. Recently, structural information has been generated from 643 SMILES sequences and applied in graph-based methods to improve overall solvation energy predictions^7^. Using the structural information, we consider three atom subsets, i.e. (A): all atoms, (B): all atoms except hydrogen, and (C): all atoms except hydrogen and carbon, for our persistent Dirac model. For (A), we generate the discrete Dirac matrices $\textbf{D}_0$ and $\textbf{D}_1$ using the Alpha complex for the filtration process from 0Å to 12Å with stepsize 0.1Å. Similarly, we generate the discrete Dirac matrices for (B) and (C) using the Rips complex for the filtration process from 0Å to 12Å with stepsize 0.1Å. We compute the 12 statistical attributes (i–xii) from the eigenvalues of $\textbf{D}_0$ and $\textbf{D}_1$ for all the 120 timesteps. From (A), (B) and (C), the total feature size sums up to $120\times 12 \times 3\times 2 = 8640$. Following the training and testing procedure in MoleculeNet^74^, we apply the random 80/10/10 split and our persistent Dirac features act as input features for a XGBoost model. Specifically, the XGBoost model is used with hyperparameters: n_estimators=20000, eta=0.1, max_depth=7, subsample=0.4, colsample_bytree=0.8. After repeating the training process 50 times and taking the mean value of the root mean squared error (RMSE) from the test predictions, our results showed that the persistent Dirac features with XGBoost achieved mean RMSE of $1.64\pm 0.31$ kcal/mol. This result is better than conventional methods from MoleculeNet^74^ as shown in Fig. 6.

Furthermore, we consider a specially-designed persistent weighted Dirac model. We define the weight of each 0-simplex (or atom) as the magnitude of electrostatic charge of the atom, weight of each 1-simplex (edge) as the Euclidean distance (in Å) between the two connected atoms and the weight of each 2-simplex (triangle) as the area of the triangle (in Å$^3$). The area of the triangle can be approximated using Heron’s formula. By generating the weighted Dirac matrices $\overline{\textbf{D}}_0$ and $\overline{\textbf{D}}_1$ along the same 120 timesteps, 12 statistical attributes (i–xii) are then computed from the eigenvalues to form our persistent weighted Dirac features. Figure 6 shows that our persistent weighted Dirac model produced a slightly lower mean RMSE of $1.62\pm 0.26$ kcal/mol as compared to the persistent Dirac model. This may suggest that the additional weight information incorporated into the weighted Dirac matrices increases the effectiveness of our approach.

## Conclusion

Molecular representations are essential to the modeling and analysis of molecular systems. Motivated by the great success of persistent Hodge Laplacian, we develop the first persistent Dirac-based molecular representation and fingerprint. A rigorous theoretical framework for persistent Dirac is introduced through the commutative diagram of discrete Dirac operator over a filtration process. Moreover, a series of persistent attributes, which characterize the persistence and variations of the eigenspectrum of Dirac matrices, are proposed and further used as molecular fingerprints. The eigenspectrum properties of discrete Dirac matrices have been studied, in particular, the geometric and topological properties of both non-homology and homology eigenvectors. We also consider weighted Dirac model and the influence of weighting schemes on eigenspectrum information. Finally, our persistent Dirac-based models have been used in the clustering of molecular configurations from nine types of organic-inorganic halide perovskites (OIHPs). This work could open new perspectives for the use of persistent Dirac-based molecular fingerprints. We hope that this can inspire future interdisciplinary work between Dirac operators and machine learning along OIHPs or other relevant research directions. An interesting direction for further exploration would be the use of non-symmetric persistent Dirac features in predicting biological, chemical and physical properties in biomolecular data. For instance, further exploration in the use of non-symmetric persistent Dirac features can be considered in the prediction of energy bandgap and other material properties in OIHPs^75^.

## Supplementary Information

Supplementary Information.

## Data Availability

The OIHP dataset generated and persistent Dirac codes during and/or analysed during the current study are available in https://github.com/ExpectozJJ/Persistent-Dirac-Models. The 3D coordinates of the molecular structures from FreeSolv database and the solvation free energy values are available in https://weilab.math.msu.edu/DataLibrary/3D/Downloads/FreeSolv.zip.
