# Supplementary material for: Persistent Dirac for molecular representation
Source: Sci Rep. 2023 Jul 11;13:11183. doi: 10.1038/s41598-023-37853-z (PMC10336089; doi:10.1038/s41598-023-37853-z)
Supplement: Supplementary file 1 — Supplementary Information. [file 41598_2023_37853_MOESM1_ESM.pdf]

Supporting Information for Persistent Dirac for molecular representation

Junjie Wee[1,*], Ginestra Bianconi[2,3,†], and Kelin Xia[1,‡]
[1]Division of Mathematical Sciences, School of Physical and Mathematical Sciences, Nanyang Technological University, Singapore 637371
[2]School of Mathematical Sciences, Queen Mary University of London, London, E1 4NS, United Kingdom
[3]The Alan Turing Institute, London, NW1 2DB, United Kingdom

June 20, 2023

Appendix A: Simplicial Complex

Formally, a p-simplex $\sigma^p = \{v_0, v_1, v_2, \cdots, v_p\}$ is defined as a convex hull formed by its $p+1$ affinely independent points $v_0, v_1, v_2, \cdots, v_p$:

$$\sigma^p = \left\{ \lambda_0 v_0 + \lambda_1 v_1 + \cdots + \lambda_p v_p \middle| \sum_{i=0}^{p} \lambda_i = 0; \forall i, 0 \leq \lambda_i \leq 1 \right\}.$$

The i-dimensional face of p-dimensional simplex σ^p (indicated with $i < p$) is the convex hull formed by $i+1$ vertices belonging to the set of $p+1$ points $\{v_0, v_1, v_2, \cdots, v_p\}$. The simplices are basic components of a simplicial complex.
A simplicial complex $\mathcal{K}$ is a finite set of simplices that satisfy two essential conditions:

- Any face of a simplex from $\mathcal{K}$ is also in $\mathcal{K}$.
- The intersection of any two simplices in $\mathcal{K}$ is either empty or formed by shared faces.

In addition, the d-skeleton of a p-dimensional simplicial complex is the simplicial complex consisting of simplices up to dimension d, where $0 \leq d \leq p$. The 1-skeleton of a simplicial complex is always the graph of simplicial complex.

Appendix B: Proofs of Elementary Properties of Upper and Lower Hodge Laplacians

The matrices $\overline{\mathbf{B}}_p$, $\overline{\mathbf{L}}_p^{\text{up}}$ and $\overline{\mathbf{L}}_p^{\text{down}}$, have various interesting properties as follows[1] (see below for proofs).

(i) $\ker \overline{\mathbf{B}}_p = \ker \overline{\mathbf{L}}_p^{\text{down}}$.

(ii) $\ker \overline{\mathbf{B}}_p^{\top} = \ker \overline{\mathbf{L}}_{p-1}^{\text{up}}$.

(iii) λ is a non-zero eigenvalue of $\overline{\mathbf{L}}_p^{\text{down}}$ with corresponding eigenvector v if and only if λ is a non-zero eigenvalue of $\overline{\mathbf{L}}_{p-1}^{\text{up}}$ with corresponding eigenvector $\overline{\mathbf{B}}_p v$. Hence, $\overline{\mathbf{L}}_{p-1}^{\text{up}}$ and $\overline{\mathbf{L}}_p^{\text{down}}$ always have the same non-zero eigenvalues.

*Address correspondences to JunJie Wee. E-mail: weej0019@e.ntu.edu.sg
†E-mail: ginestra.bianconi@gmail.com
‡E-mail: xiakelin@ntu.edu.sg

(iv) $v \in \ker \overline{\mathbf{L}}_p^{\text{down}}$ if and only if $\overline{\mathbf{B}}_p v \in \ker \overline{\mathbf{L}}_{p-1}^{\text{up}}$.

(v) $\operatorname{im} \overline{\mathbf{L}}_p^{\text{up}} \subset \ker \overline{\mathbf{L}}_p^{\text{down}}$.

(vi) $\operatorname{im} \overline{\mathbf{L}}_p^{\text{down}} \subset \ker \overline{\mathbf{L}}_p^{\text{up}}$.

(vii) $\ker \overline{\mathbf{B}}_p^\top = (\operatorname{im} \overline{\mathbf{B}}_p)^\perp$

Proof. (i) For any $v \in \ker \overline{\mathbf{L}}_p^{\text{down}}$, v satisfies $\overline{\mathbf{L}}_p^{\text{down}} v = \mathbf{0}$, then $v^\top \overline{\mathbf{B}}_p^\top \overline{\mathbf{B}}_p v = v^\top \overline{\mathbf{L}}_p^{\text{down}} v = \mathbf{0}$ which shows that $\overline{\mathbf{B}}_p v = \mathbf{0}$. Hence, $v \in \ker \overline{\mathbf{B}}_p$. On the other hand, for any $v \in \ker \overline{\mathbf{B}}_p$, we have $\overline{\mathbf{B}}_p v = \mathbf{0}$. Multiplying both sides by $\overline{\mathbf{B}}_p^\top$ implies that $\overline{\mathbf{B}}_p^\top \overline{\mathbf{B}}_p v = \mathbf{0}$.

(ii) For any $v \in \ker \overline{\mathbf{L}}_{p-1}^{\text{up}}$, v satisfies $\overline{\mathbf{L}}_{p-1}^{\text{up}} v = \mathbf{0}$, then $v^\top \overline{\mathbf{B}}_p \overline{\mathbf{B}}_p^\top v = \mathbf{0}$ which shows that $\overline{\mathbf{B}}_p^\top v = \mathbf{0}$. Hence, $v \in \ker \overline{\mathbf{B}}_p^\top$. On the other hand, for any $v \in \ker \overline{\mathbf{B}}_p^\top$, we have $\overline{\mathbf{B}}_p^\top v = \mathbf{0}$. Multiplying both sides by $\overline{\mathbf{B}}_p$ implies that $\overline{\mathbf{B}}_p \overline{\mathbf{B}}_p^\top v = \mathbf{0}$.

(iii) Since

$$\begin{aligned} \overline{\mathbf{L}}_p^{\text{down}} v = \lambda v &\iff \overline{\mathbf{B}}_p \overline{\mathbf{B}}_p^\top \overline{\mathbf{B}}_p v = \lambda \overline{\mathbf{B}}_p v \\ &\iff \overline{\mathbf{L}}_{p-1}^{\text{up}} \overline{\mathbf{B}}_p v = \lambda \overline{\mathbf{B}}_p v, \end{aligned}$$

then λ is a non-zero eigenvalue of $\overline{\mathbf{L}}_p^{\text{down}}$ with corresponding eigenvector $\overline{\mathbf{B}}_p v$.

(iv) Similar to (iii), $\overline{\mathbf{L}}_p^{\text{down}} v = 0 \iff \overline{\mathbf{L}}_{p-1}^{\text{up}} \overline{\mathbf{B}}_p v = 0$.

(v) For any $v \in \operatorname{im} \overline{\mathbf{L}}_p^{\text{up}}$, there exist some w such that $\overline{\mathbf{L}}_p^{\text{up}} w = v$. Hence,

$$\begin{aligned} \overline{\mathbf{L}}_p^{\text{down}} v = \overline{\mathbf{B}}_p^\top \overline{\mathbf{B}}_p v &= \overline{\mathbf{B}}_p^\top \overline{\mathbf{B}}_p \overline{\mathbf{L}}_p^{\text{up}} w \\ &= \overline{\mathbf{B}}_p^\top \underbrace{\overline{\mathbf{B}}_p \overline{\mathbf{B}}_{p+1}}_{=\mathbf{0}} \overline{\mathbf{B}}_{p+1}^\top w = \mathbf{0}. \end{aligned}$$

(vi) For any $v \in \operatorname{im} \overline{\mathbf{L}}_p^{\text{down}}$, there exist some w such that $\overline{\mathbf{L}}_p^{\text{down}} w = v$. Hence,

$$\begin{aligned} \overline{\mathbf{L}}_p^{\text{up}} v = \overline{\mathbf{B}}_{p+1} \overline{\mathbf{B}}_{p+1}^\top v &= \overline{\mathbf{B}}_{p+1} \overline{\mathbf{B}}_{p+1}^\top \overline{\mathbf{L}}_p^{\text{down}} w \\ &= \overline{\mathbf{B}}_{p+1} \underbrace{\overline{\mathbf{B}}_{p+1}^\top \overline{\mathbf{B}}_p^\top}_{=\mathbf{0}} \overline{\mathbf{B}}_p w = \mathbf{0}. \end{aligned}$$

(vii) Define the orthogonal complement

$$\ker(\overline{\mathbf{B}}_p)^\perp = \{c \in C_p | c \perp d, \quad \forall d \in \ker(\overline{\mathbf{B}}_p)\}.$$

Note that $\ker(\overline{\mathbf{B}}_p)^\perp = \operatorname{im}(\overline{\mathbf{B}}_p^\top)$. This is easily seen since for any $c \in \ker(\overline{\mathbf{B}}_p)^\perp$, we have $\overline{\mathbf{B}}_p(c) = d \neq 0$ and hence

$$\overline{\mathbf{B}}_p^\top \overline{\mathbf{B}}_p(c) = \overline{\mathbf{B}}_p^\top (d) = c.$$

Similarly, $\overline{\mathbf{B}}_p(c) = \overline{\mathbf{B}}_p \overline{\mathbf{B}}_p^\top (d) = d$.

By replacing $\overline{\mathbf{B}}_p$ with $\overline{\mathbf{B}}_p^\top$ and $\overline{\mathbf{B}}_p^\top$ with $\overline{\mathbf{B}}_p$, one obtains

$$\ker(\overline{\mathbf{B}}_p^\top)^\perp = \operatorname{im}(\overline{\mathbf{B}}_p).$$

Lastly, by taking orthogonal complement on both sides,

$$\ker \overline{\mathbf{B}}_p^\top = (\operatorname{im} \overline{\mathbf{B}}_p)^\perp.$$

□

Appendix C: Supplementary Details About Hodge Laplacian

In this appendix, we show that (a): $\dim\ker\overline{\mathbf{L}}_p$ can be rewritten as $\dim\ker\overline{\mathbf{L}}_p^{\text{down}} - \dim\operatorname{im}\overline{\mathbf{L}}_p^{\text{up}}$ and in (b): Any eigenvector v of $\overline{\mathbf{L}}_p$ can only either be $v \in \operatorname{im}\overline{\mathbf{L}}_p^{\text{up}} \subset \ker\overline{\mathbf{L}}_p^{\text{down}}$ or $v \in \operatorname{im}\overline{\mathbf{L}}_p^{\text{down}} \subset \ker\overline{\mathbf{L}}_p^{\text{up}}$.

Proof. (a): Note that $\dim\ker\overline{\mathbf{L}}_p = \beta_p$ is similarly proven by Eckmann in 1944.[2] Hence,

$$\begin{aligned}
\dim\ker\overline{\mathbf{L}}_p = \beta_p &= \text{rank } Z_p - \text{rank } B_p \\
&= \dim\ker\overline{\mathbf{B}}_p - \text{rank } \overline{\mathbf{B}}_{p+1} \\
&= \dim\ker\overline{\mathbf{L}}_p^{\text{down}} - \text{rank } \overline{\mathbf{B}}_{p+1}^{\top} \\
&= \dim\ker\overline{\mathbf{L}}_p^{\text{down}} - \dim C_p + \dim\ker\overline{\mathbf{B}}_{p+1}^{\top} \\
&= \dim\ker\overline{\mathbf{L}}_p^{\text{down}} - \dim C_p + \dim\ker\overline{\mathbf{L}}_p^{\text{up}} \\
&= \dim\ker\overline{\mathbf{L}}_p^{\text{down}} - \dim\operatorname{im}\overline{\mathbf{L}}_p^{\text{up}}.
\end{aligned}$$

(b): ($\Longleftarrow$): For any non-zero eigenvalue λ of $\overline{\mathbf{L}}_p^{\text{down}}$ (resp. $\overline{\mathbf{L}}_p^{\text{up}}$) with eigenvector v, (iv) and (v) (Appendix B) shows that $v \in \operatorname{im}\overline{\mathbf{L}}_p^{\text{up}}$ (resp. $v \in \operatorname{Im}\overline{\mathbf{L}}_p^{\text{down}}$). Hence, $v \in \ker\overline{\mathbf{L}}_p^{\text{down}}$ or $v \in \ker\overline{\mathbf{L}}_p^{\text{up}}$. Then for both cases,

$$\overline{\mathbf{L}}_p v = (\overline{\mathbf{L}}_p^{\text{down}} + \overline{\mathbf{L}}_p^{\text{up}})v = \overline{\mathbf{L}}_p^{\text{down}} v + \overline{\mathbf{L}}_p^{\text{up}} v = \lambda v.$$

($\Longrightarrow$): For any non-zero eigenvalue λ of $\overline{\mathbf{L}}_p$,

$$\overline{\mathbf{L}}_p v = \lambda v \implies \overline{\mathbf{L}}_p^{\text{down}} v + \overline{\mathbf{L}}_p^{\text{up}} v = \lambda v.$$

From (v) and (vi) (Appendix B), a similar argument follows by showing that either $v \in \operatorname{im}\overline{\mathbf{L}}_p^{\text{up}} \subset \ker\overline{\mathbf{L}}_p^{\text{down}}$ or $v \in \operatorname{im}\overline{\mathbf{L}}_p^{\text{down}} \subset \ker\overline{\mathbf{L}}_p^{\text{up}}$. □

Additionally, let $\mathbf{s}(\overline{\mathbf{L}}_p^{\text{up}})$ and $\mathbf{s}(\overline{\mathbf{L}}_p^{\text{down}})$ be the spectrum of $\overline{\mathbf{L}}_p^{\text{up}}$ and $\overline{\mathbf{L}}_p^{\text{down}}$ respectively. Suppose the highest order of the simplicial complex $\mathcal{K}$ is d. Similar to,[1] the multiplicity of zero eigenvalues in

(i) $\mathbf{s}(\overline{\mathbf{L}}_p^{\text{up}})$ can be computed as

$$\dim C_p - \sum_{i=0}^{p} (-1)^{i+p} (\dim C_i - \dim H_i), \tag{1}$$

(ii) $\mathbf{s}(\overline{\mathbf{L}}_p^{\text{down}})$ can be computed as

$$\dim C_p - \sum_{i=0}^{p-1} (-1)^{p-1+i} (\dim C_i - \dim H_i). \tag{2}$$

Proof. (i): From Appendix B,

$$\begin{aligned}
\dim\ker\overline{\mathbf{L}}_p^{\text{up}} &= \dim\ker\overline{\mathbf{B}}_{p+1}^{\top} \\
&= \dim C_p - \dim\operatorname{im}\overline{\mathbf{B}}_{p+1}^{\top} \\
&= \dim C_p - \dim\operatorname{im}\overline{\mathbf{B}}_{p+1}.
\end{aligned}$$

Then

$$\begin{aligned}
\dim\operatorname{im}\overline{\mathbf{B}}_{p+1} &= \dim\ker\overline{\mathbf{B}}_p - \dim H_p \\
&= \dim C_p - \dim H_p - \dim\operatorname{im}\overline{\mathbf{B}}_p \\
&= \cdots = \sum_{i=0}^{p} (-1)^{i+p} (\dim C_i - \dim H_i).
\end{aligned}$$

Putting everything together yields

$$\begin{aligned}\mathbf{s}(\overline{\mathbf{L}}_p^{\text{up}}) &= \dim C_p - \dim \text{im } \overline{\mathbf{B}}_{p+1} \\ &= \dim C_p - \sum_{i=0}^{p} (-1)^{i+p} (\dim C_i - \dim H_i).\end{aligned}$$

(ii): Since $\mathbf{s}(\overline{\mathbf{L}}_p^{\text{down}}) = \mathbf{s}(\overline{\mathbf{B}}_p) = \dim C_p - \dim \text{im } \overline{\mathbf{B}}_p$, then

$$\begin{aligned}\dim \text{im } \overline{\mathbf{B}}_p &= \dim \ker \overline{\mathbf{B}}_{p-1} - \dim H_{p-1} \\ &= \dim C_{p-1} - \dim H_{p-1} - \dim \text{im } \overline{\mathbf{B}}_{p-1} \\ &= \cdots = \sum_{i=0}^{p-1} (-1)^{i+p-1} (\dim C_i - \dim H_i).\end{aligned}$$

Therefore,

$$\begin{aligned}\mathbf{s}(\overline{\mathbf{L}}_p^{\text{down}}) &= \dim C_p - \dim \text{im } \overline{\mathbf{B}}_p \\ &= \dim C_p - \sum_{i=0}^{p-1} (-1)^{i+p-1} (\dim C_i - \dim H_i).\end{aligned}$$

□

Appendix D: Proof for Hodge Decomposition

Recall from Section **??** that $\dim \ker \overline{\mathbf{L}}_p^{\text{down}} = \beta_p + \text{rank } \overline{\mathbf{B}}_{p+1}^\top$. This means that the number of zero eigenvalues in $\overline{\mathbf{L}}_p^{\text{down}}$ is equal to the sum of rank $\overline{\mathbf{B}}_{p+1}^\top$ and β_p. Furthermore, since $H_p = Z_p/B_p = \ker \overline{\mathbf{B}}_p / \text{im } \overline{\mathbf{B}}_{p+1}$, then

$$\ker \overline{\mathbf{L}}_p \cong H_p = \ker \overline{\mathbf{B}}_p \cap (\text{im } \overline{\mathbf{B}}_{p+1})^\perp.$$

Here, $(\text{im } \overline{\mathbf{B}}_{p+1})^\perp$ is the orthogonal complement of im $\overline{\mathbf{B}}_{p+1}$. In fact, $(\text{im } \overline{\mathbf{B}}_{p+1})^\perp = \ker \overline{\mathbf{B}}_{p+1}^\top$. This is true because from (v), $\ker \overline{\mathbf{B}}_p^\top = (\text{im } \overline{\mathbf{B}}_p)^\perp$ (see Appendix B for the proof). Hence, this gives

$$\ker \overline{\mathbf{B}}_p \cap (\text{im } \overline{\mathbf{B}}_{p+1})^\perp = \ker \overline{\mathbf{B}}_p \cap \ker \overline{\mathbf{B}}_{p+1}^\top.$$

In other words, we have

$$\ker \overline{\mathbf{L}}_p = \ker \overline{\mathbf{B}}_p \cap \ker \overline{\mathbf{B}}_{p+1}^\top.$$

This means that for every $v \in \ker \overline{\mathbf{L}}_p$,

$$v \in \ker \overline{\mathbf{B}}_p \cap \ker \overline{\mathbf{B}}_{p+1}^\top.$$

Proof. Recall from Appendix A that $\ker(\overline{\mathbf{B}}_p)^\perp = \text{im}(\overline{\mathbf{B}}_p^\top)$. Hence,

$$\begin{aligned}C_p &= \ker(\overline{\mathbf{B}}_p) \oplus \ker(\overline{\mathbf{B}}_p)^\perp \\ &= \ker(\overline{\mathbf{B}}_p) \oplus \text{im}(\overline{\mathbf{B}}_p^\top) \\ &= \text{im}(\overline{\mathbf{B}}_{p+1}) \oplus \ker(\overline{\mathbf{L}}_p) \oplus \text{im}(\overline{\mathbf{B}}_p^\top),\end{aligned}$$

since $\ker(\overline{\mathbf{L}}_p) = H_p = \ker(\overline{\mathbf{B}}_p) / \text{im}(\overline{\mathbf{B}}_{p+1})$. □

The vector space of edge flows C_1 admits the following orthogonal sum decomposition in Helmholtz-Hodge Decomposition:

$$C_1 = \text{im}(\text{curl}^*) \oplus \ker(\overline{\mathbf{L}}_1) \oplus \text{im}(\text{grad}),$$

where

$$\ker(\overline{\mathbf{L}}_1) = \ker(\text{curl}) \cap \ker(\text{div}).$$

Appendix E: Proof that λ^s is eigenvalue of Dirac operator

Proof. Since

$$\mathbf{D}_p v = \lambda v, \tag{3}$$

then multiplying by $\mathbf{D}_p^{s-1}$ gives

$$\begin{aligned}\mathbf{D}_p^s v &= \lambda \mathbf{D}_p^{s-1} v \\ &= \lambda \mathbf{D}_p^{s-2} \mathbf{D}_p v \\ &= \lambda^2 \mathbf{D}_p^{s-2} v = \cdots = \lambda^s v. \quad \text{(By (3))}\end{aligned}$$

Similarly, since $\mathbf{D}_p v = \lambda v$, we also have

$$\mathbf{D}_p \mathbf{Q}_p v = -\lambda \mathbf{Q}_p v. \tag{4}$$

Hence,

$$\begin{aligned}\mathbf{D}_p^s \mathbf{Q}_p v &= -\lambda \mathbf{D}_p^{s-1} \mathbf{Q}_p v \\ &= -\lambda \mathbf{D}_p^{s-2} \mathbf{D}_p \mathbf{Q}_p v \\ &= \lambda^2 \mathbf{D}_p^{s-2} \mathbf{Q}_p v = \cdots = (-\lambda)^s \mathbf{Q}_p v. \quad \text{(By (4))}\end{aligned}$$

□

Appendix F: Additional DO-based Fingerprints of OIHPs

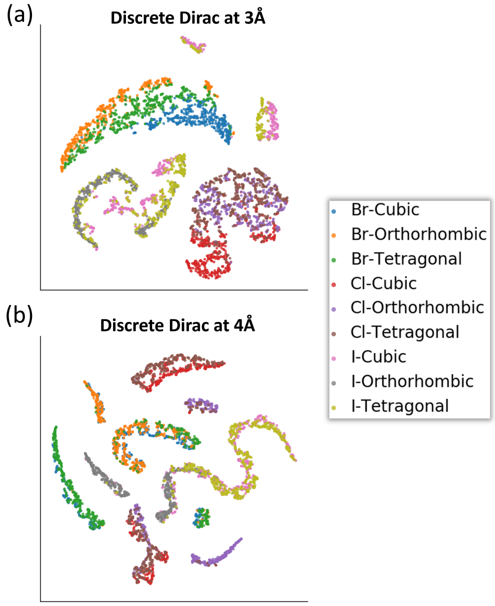

Figure S1: The clustering of 9 types of OIHP molecular dynamics (MD) trajectories using DO-based features at other filtration times, namely, (A) Dirac operator at 3Å and 4Å. However, without persistence, the Dirac operators at specific cutoff distances only provide certain topological information. The x-axis and y-axis are the two principal components obtained from the t-SNE model.

Appendix G: Persistent Multiplicities of Hodge Laplacians

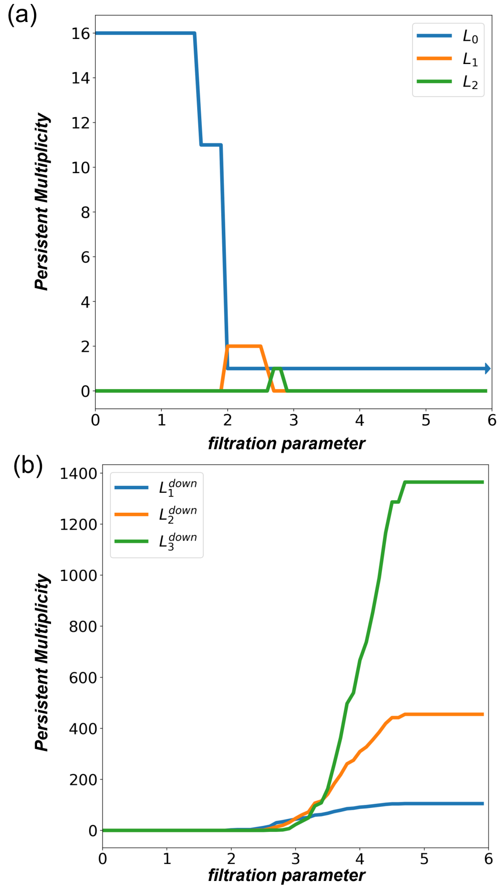

Figure S2: The clustering of 9 types of OIHP molecular dynamics (MD) trajectories using DO-based features at other filtration times, namely, (A) Dirac operator at 3Å and 4Å. However, without persistence, the Dirac operators at specific cutoff distances only provide certain topological information. The x-axis and y-axis are the two principal components obtained from the t-SNE model.

Appendix H: Spectrum of Dirac Matrices

Let $\mathbf{A}_1$ be an $m_1 \times n_1$ matrix and $\mathbf{A}_2$ to be a $m_2 \times n_2$ matrix,

$$\mathbf{diag}(\mathbf{A}_1, \mathbf{A}_2) = \begin{bmatrix} \mathbf{A}_1 & \mathbf{0}_{m_1 \times n_2} \\ \mathbf{0}_{m_2 \times n_1} & \mathbf{A}_2 \end{bmatrix}.$$

This gives

$$\begin{aligned} \mathbf{D}_p^2 &= \mathbf{diag}(\mathbf{L}_0, \mathbf{L}_1, \cdots, \mathbf{L}_{p+1}) \\ &= \mathbf{diag}(\mathbf{L}_0^{\text{up}}, \mathbf{L}_1^{\text{up}}, \cdots, \mathbf{L}_{p+1}^{\text{up}}) \\ &\quad + \mathbf{diag}(\mathbf{L}_0^{\text{down}}, \mathbf{L}_1^{\text{down}}, \cdots, \mathbf{L}_{p+1}^{\text{down}}), \end{aligned}$$

where $\mathbf{L}_{p+1}^{\text{up}} = \mathbf{0}_{n_{p+1} \times n_{p+1}}$ and $\mathbf{L}_0^{\text{down}} = \mathbf{0}_{n_0 \times n_0}$. For convenience, we simply denote $\mathbf{diag}(\mathbf{L}_0^{\text{up}}, \mathbf{L}_1^{\text{up}}, \cdots, \mathbf{L}_{p+1}^{\text{up}})$ as $(\mathbf{D}_p^2)^{\text{up}}$ and $\mathbf{diag}(\mathbf{L}_0^{\text{down}}, \mathbf{L}_1^{\text{down}}, \cdots, \mathbf{L}_{p+1}^{\text{down}})$ as $(\mathbf{D}_p^2)^{\text{down}}$.
Let $\mathbf{s}(\mathbf{D}_p^2)^{\text{up}}$ and $\mathbf{s}(\mathbf{D}_p^2)^{\text{down}}$ be the spectrum of the upper and lower $\mathbf{D}_p^2$. Following (1) and (2), the multiplicity of zero eigenvalues for

(i) $\mathbf{s}(\mathbf{D}_p^2)^{\text{up}}$ can be computed as

$$\sum_{k=0}^{p+1}\left(\dim C_k - \sum_{i=0}^{k}(-1)^{i+k}(\dim C_i - \dim H_i)\right).$$

(ii) $\mathbf{s}(\mathbf{D}_p^2)^{\text{down}}$ can be computed as

$$\sum_{k=0}^{p+1}\left(\dim C_k - \sum_{i=0}^{k-1}(-1)^{k-1+i}(\dim C_i - \dim H_i).\right).$$

Furthermore, we also have

$$\begin{aligned}\dim\ker \mathbf{D}_p^2 &= \sum_{k=0}^{p+1}\beta_k = \sum_{k=0}^{p+1}(\dim\ker \mathbf{L}_k^{\text{down}} - \dim\operatorname{im}\mathbf{L}_k^{\text{up}})\\ &= \sum_{k=0}^{p+1}\dim\ker \mathbf{L}_k^{\text{down}} - \sum_{k=0}^{p+1}\dim\operatorname{im}\mathbf{L}_k^{\text{up}}\\ &= \dim\ker(\mathbf{D}_p^2)^{\text{up}} - \dim\operatorname{im}(\mathbf{D}_p^2)^{\text{down}}.\end{aligned}$$

Appendix I: Derivation of the real spectrum of the Weighted Dirac matrix

By setting $\mathbf{G}_n$ as identity matrices, we obtain the Dirac matrices whose eigenvalues has been shown to be always real.[3] This special case is discussed and has been applied to signal processing by proposing the use of topological spinors obtained from eigenspectrum of Dirac matrices.[3] Essentially, an n-dimensional topological spinor $\mathbf{s}$ can be written as

$$\mathbf{s} = \begin{bmatrix}\mathbf{s}_0\\ \mathbf{s}_1\\ \vdots\\ \mathbf{s}_n\end{bmatrix} \in \mathcal{C}_n,$$

where $\mathcal{C}_n$ is the space of all n-dimensional topological spinors. Here, the n-dimensional topological spinor $\mathbf{s}$ is a direct sum of block vectors (signals) $\mathbf{s}_k$ defined for k-simplices, $0 \le k \le n$. Now, we shall provide a similar treatment to the weighted Dirac matrices which may or may not be symmetric but can be shown to always have real eigenvalues. To be concrete we will focus on the case in which the simplicial complex is two dimensional, i.e. formed by nodes, links and triangles. Extension of these results to higher-order Dirac operators is straightforward.

Recall from the definition of weighted Dirac matrix that $\overline{\mathbf{D}}_1$ is written as

$$\overline{\mathbf{D}}_1 = \begin{bmatrix}\mathbf{0}_{n_0\times n_0} & \mathbf{G}_0^{-1}\mathbf{B}_1\mathbf{G}_1/\sqrt{2} & \mathbf{0}_{n_0\times n_2}\\ \mathbf{B}_1^\top/\sqrt{2} & \mathbf{0}_{n_1\times n_1} & \mathbf{G}_1^{-1}\mathbf{B}_2\mathbf{G}_2/\sqrt{3}\\ \mathbf{0}_{n_2\times n_0} & \mathbf{B}_2^\top/\sqrt{3} & \mathbf{0}_{n_2\times n_2}\end{bmatrix}$$

We shall write $\overline{\mathbf{D}}_1$ as $\overline{\mathbf{D}}_{[0]} + \overline{\mathbf{D}}_{[1]}$ where

$$\overline{\mathbf{D}}_{[0]} = \begin{bmatrix}\mathbf{0}_{n_0\times n_0} & \mathbf{G}_0^{-1}\mathbf{B}_1\mathbf{G}_1/\sqrt{2} & \mathbf{0}_{n_0\times n_2}\\ \mathbf{B}_1^\top/\sqrt{2} & \mathbf{0}_{n_1\times n_1} & \mathbf{0}_{n_1\times n_2}\\ \mathbf{0}_{n_2\times n_0} & \mathbf{0}_{n_2\times n_1} & \mathbf{0}_{n_2\times n_2}\end{bmatrix}$$

and

$$\overline{\mathbf{D}}_{[1]} = \begin{bmatrix} \mathbf{0}_{n_0\times n_0} & \mathbf{0}_{n_0\times n_1} & \mathbf{0}_{n_0\times n_2} \\ \mathbf{0}_{n_1\times n_0} & \mathbf{0}_{n_1\times n_1} & \mathbf{G}_1^{-1}\mathbf{B}_2\mathbf{G}_2/\sqrt{3} \\ \mathbf{0}_{n_2\times n_0} & \mathbf{B}_2^\top/\sqrt{3} & \mathbf{0}_{n_2\times n_2} \end{bmatrix}$$

Note that $\overline{\mathbf{D}}_{[1]}\overline{\mathbf{D}}_{[0]} = \mathbf{0}$ and $\overline{\mathbf{D}}_{[0]}\overline{\mathbf{D}}_{[1]} = \mathbf{0}$, which implies that

$$\text{im } \overline{\mathbf{D}}_{[1]} \subseteq \ker \overline{\mathbf{D}}_{[0]}, \quad \text{im } \overline{\mathbf{D}}_{[0]} \subseteq \ker \overline{\mathbf{D}}_{[1]}.$$

This means that the weighted Dirac matrix $\overline{\mathbf{D}}_1$ admits the following Dirac decomposition:[3]

$$\mathcal{C}_2 = \ker \overline{\mathbf{D}}_1 \oplus \text{im } \overline{\mathbf{D}}_{[0]} \oplus \text{im } \overline{\mathbf{D}}_{[1]},$$

where

$$\ker \overline{\mathbf{D}}_1 = \ker \overline{\mathbf{L}}_{[0]} \oplus \ker \overline{\mathbf{L}}_{[1]} \oplus \ker \overline{\mathbf{L}}_{[2]}.$$

The above Dirac decomposition implies that the non-zero eigenvectors of $\overline{\mathbf{D}}_1$ are either non-zero eigenvectors of $\overline{\mathbf{D}}_{[0]}$ (corresponding to an eigenvalue λ_0) or non zero eigenvectors of $\overline{\mathbf{D}}_{[1]}$ (corresponding to an eigenvalue λ_1). Here, define the matrix Φ of the eigenvectors of $\overline{\mathbf{D}}_1$ as

$$\mathbf{\Phi} = \begin{bmatrix} \mathbf{\Phi}_0 & \mathbf{\Phi}_1 & \mathbf{\Phi}_{\text{harm}} \end{bmatrix}.$$

where $\mathbf{\Phi}_n$ is the matrix of the eigenvectors $\boldsymbol{\phi}_n \in \text{im}(\overline{\mathbf{D}}_{[n]})$ with $n \in \{0, 1\}$ and $\mathbf{\Phi}_{\text{harm}}$ is the matrix of eigenvectors forming a basis for $\ker(\overline{\mathbf{D}}_1)$.
Now, denote $\mathbf{u}_0$ as the eigenvector of $\mathbf{L}_{[0]}$ and $\mathbf{v}_0$ as the eigenvector of $\mathbf{L}_{[1]}^{\text{down}}$ corresponding to the same non zero eigenvalue Λ_0, i.e. satisfying the relations

$$\mathbf{L}_{[0]}\mathbf{u}_0 = \Lambda_0\mathbf{u}_0, \quad \mathbf{L}_{[1]}^{\text{down}}\mathbf{v}_0 = \Lambda_0\mathbf{v}_0$$

and similarly, we have $\mathbf{v}_1$ as the eigenvector of $\mathbf{L}_{[1]}^{\text{up}}$ and $\mathbf{z}_1$ as the eigenvector of $\mathbf{L}_{[2]}^{\text{down}}$ corresponding to a same non zero eigenvalue Λ_1, i.e. satisfying the relations

$$\mathbf{L}_{[1]}^{\text{up}}\mathbf{v}_1 = \Lambda_1\mathbf{v}_1, \quad \mathbf{L}_{[2]}^{\text{down}}\mathbf{z}_1 = \Lambda_1\mathbf{z}_1,$$

with $\mathbf{u}_0, \mathbf{v}_0, \mathbf{v}_1$ and $\mathbf{z}_1$ being eigenvectors normalized to one. Using a notation from Appendix H, we can also write $\overline{\mathbf{D}}_p^2 = \mathbf{diag}(\mathbf{L}_{[0]}, \mathbf{L}_{[1]}, \cdots, \mathbf{L}_{[p+1]})$. This implies that eigenvectors $\overline{\mathbf{D}}_{[0]}$ and the eigenvectors of $\overline{\mathbf{D}}_{[1]}$ takes the form

$$\mathbf{\Phi}_0 = \begin{bmatrix} \mathbf{U}_0 & \mathbf{U}_0 \\ \mathbf{V}_0 & -\mathbf{V}_0 \\ \mathbf{0} & \mathbf{0} \end{bmatrix}, \mathbf{\Phi}_1 = \begin{bmatrix} \mathbf{0} & \mathbf{0} \\ \mathbf{V}_1 & \mathbf{V}_1 \\ \mathbf{Z}_1 & -\mathbf{Z}_1 \end{bmatrix}$$

where $\mathbf{U}_0, \mathbf{V}_0, \mathbf{V}_1, \mathbf{Z}_1$ are the matrices formed by vectors proportional to the eigenvectors $\mathbf{u}_0, \mathbf{v}_0, \mathbf{v}_1$ and $\mathbf{z}_1$ respectively. In particular we have that the eigenvectors $\boldsymbol{\phi}_{\boldsymbol{n}}$ with $n \in \{0, 1\}$ can be written as

$$\boldsymbol{\phi}_0^+ = \frac{1}{\mathcal{N}_0}\begin{pmatrix} \mathbf{u}_0 \\ \mathbf{v}_0 \\ \mathbf{0} \end{pmatrix}, \quad \boldsymbol{\phi}_0^- = \frac{1}{\mathcal{N}_0}\begin{pmatrix} \mathbf{u}_0 \\ -\mathbf{v}_0 \\ \mathbf{0} \end{pmatrix}$$

$$\boldsymbol{\phi}_1^+ = \frac{1}{\mathcal{N}_1}\begin{pmatrix} \mathbf{0} \\ \mathbf{v}_1 \\ \mathbf{z}_1 \end{pmatrix}, \quad \boldsymbol{\phi}_1^- = \frac{1}{\mathcal{N}_1}\begin{pmatrix} \mathbf{0} \\ \mathbf{v}_1 \\ -\mathbf{z}_1 \end{pmatrix}.$$

Let us indicate with $\mathbf{u}_{\rm harm}, \mathbf{v}_{\rm harm}$ and $\boldsymbol{z}_{\rm harm}$ the eigenvectors corresponding to the zero eigenvalue of $\mathbf{L}_{[0]}, \mathbf{L}_{[1]}$ and $\mathbf{L}_{[2]}$ respectively, i.e. satisfying

$$\begin{aligned}\mathbf{L}_{[0]}\mathbf{u}_{\rm harm} &= \mathbf{0},\\ \mathbf{L}_{[1]}\mathbf{v}_{\rm harm} &= (\mathbf{L}_{[1]}^{\rm up}+\mathbf{L}_{[1]}^{\rm down})\mathbf{v}_{\rm harm} = \mathbf{0},\\ \mathbf{L}_{[2]}\mathbf{z}_{\rm harm} &= \mathbf{0}.\end{aligned}$$

We have that

$$\boldsymbol{\Phi}_{\rm harm} = \begin{bmatrix} \boldsymbol{U}_{\rm harm} & \mathbf{0} & \mathbf{0} \\ \mathbf{0} & \boldsymbol{V}_{\rm harm} & \mathbf{0} \\ \mathbf{0} & \mathbf{0} & \boldsymbol{Z}_{\rm harm}\end{bmatrix},$$

where $\boldsymbol{U}_{\rm harm}, \boldsymbol{V}_{\rm harm}$ and $\boldsymbol{Z}_{\rm harm}$ are the matrices of eigenvectors $\mathbf{u}_{\rm harm}, \mathbf{v}_{\rm harm}$ and $\mathbf{z}_{\rm harm}$ respectively. The weighted Dirac matrix has eigenvalues which can be null, positive or negative. The positive part of the spectrum is given by the square root of the eigenvectors of the (normalized) Hodge Laplacian and for each positive eigenvector there is a negative eigenvector with the same absolute value. The eigenvectors of the weighted Dirac matrix are formed by the direct sum of the eigenvectors of the weighted Hodge Laplacians. The normalized weighted Dirac matrix has positive, zero and negative eigenvalues λ_n that have absolute value smaller or equal to one[3]

$$|\lambda_n| \leq 1.$$

with λ_n related to the eigenvalues Λ_{n-1} by

$$\lambda_n = \pm\sqrt{\Lambda_{n-1}}. \tag{5}$$

Now let us define

$$\tilde{\mathbf{B}}_n = \mathbf{G}_{n-1}^{-1/2}\mathbf{B}_n\mathbf{G}_n^{1/2}/\sqrt{n+1}$$

and equivalently its transpose

$$\tilde{\mathbf{B}}_n^{\top} = \mathbf{G}_n^{1/2}\mathbf{B}_n^{\top}\mathbf{G}_{n-1}^{-1/2}/\sqrt{n+1}.$$

whose product leads to the symmetric normalized Hodge Laplacians $\tilde{\mathbf{L}}_{[n-1]}^{\rm up} = \tilde{\mathbf{B}}_n\tilde{\mathbf{B}}_n^{\top}$ and $\tilde{\mathbf{L}}_{[n]}^{\rm down} = \tilde{\mathbf{B}}_n^{\top}\tilde{\mathbf{B}}_n$. From this definition it follows that

$$\begin{aligned}\tilde{\mathbf{L}}_{[n-1]}^{\rm up} &= \mathbf{G}_{n-1}^{1/2}\mathbf{L}_{[n]}^{\rm up}\mathbf{G}_{n-1}^{-1/2}\\ \tilde{\mathbf{L}}_{[n]}^{\rm down} &= \mathbf{G}_n^{1/2}\mathbf{L}_{[n]}^{\rm down}\mathbf{G}_n^{-1/2}\end{aligned} \tag{6}$$

By defining $\tilde{\Lambda}_n$ as the eigenvalues satisfying

$$\begin{aligned}\tilde{\mathbf{L}}_{[0]}\tilde{\mathbf{u}}_0 = \tilde{\Lambda}_0\tilde{\mathbf{u}}_0, \quad \tilde{\mathbf{L}}_{[1]}^{\rm down}\tilde{\mathbf{v}}_0 = \tilde{\Lambda}_0\tilde{\mathbf{v}}_0\\ \tilde{\mathbf{L}}_{[1]}^{\rm up}\tilde{\mathbf{v}}_1 = \tilde{\Lambda}_1\tilde{\mathbf{v}}_2, \quad \tilde{\mathbf{L}}_{[2]}^{\rm down}\tilde{\mathbf{z}}_1 = \Lambda_1\tilde{\mathbf{z}}_1,\end{aligned} \tag{7}$$

It is easy to show that the eigenvalues Λ_n are equal to the eigenvalues $\tilde{\Lambda}_n$, i.e.

$$\Lambda_n = \tilde{\Lambda}_n = |\mu_n|^2$$

where μ_n is the singular value of $\tilde{\mathbf{B}}_n$ and that

$$\begin{aligned}\mathbf{u}_0 = \mathbf{G}_0^{-1/2}\tilde{\mathbf{u}}_0 \quad \mathbf{v}_0 = \mathbf{G}_1^{-1/2}\tilde{\mathbf{v}}_0\\ \mathbf{v}_1 = \mathbf{G}_1^{-1/2}\tilde{\mathbf{v}}_1 \quad \mathbf{z}_1 = \mathbf{G}_2^{-1/2}\tilde{\mathbf{z}}_1.\end{aligned} \tag{8}$$

It follows that the spectrum of the normalized Dirac operator is real although the operator is not symmetric with

$$\lambda_n = \pm|\mu_n| \tag{9}$$

and the eigenvectors $\boldsymbol{\phi}_0^{\pm}$ and $\boldsymbol{\phi}_1^{\pm}$ are given by

$$\boldsymbol{\phi}_0^{+} = \frac{1}{\mathcal{N}_0}\left(\begin{array}{c} \mathbf{G}_0^{-1/2}\tilde{\mathbf{u}}_0 \\ \mathbf{G}_1^{-1/2}\tilde{\mathbf{v}}_0 \\ \mathbf{0} \end{array}\right), \quad \boldsymbol{\phi}_0^{-} = \frac{1}{\mathcal{N}_0}\left(\begin{array}{c} \mathbf{G}_0^{-1/2}\tilde{\mathbf{u}}_0 \\ -\mathbf{G}_1^{-1/2}\tilde{\mathbf{v}}_0 \\ \mathbf{0} \end{array}\right)$$

$$\boldsymbol{\phi}_1^{+} = \frac{1}{\mathcal{N}_1}\left(\begin{array}{c} \mathbf{0} \\ \mathbf{G}_1^{-1/2}\tilde{\mathbf{v}}_1 \\ \mathbf{G}_2^{-1/2}\tilde{\mathbf{z}}_1 \end{array}\right), \quad \boldsymbol{\phi}_1^{-} = \frac{1}{\mathcal{N}_1}\left(\begin{array}{c} \mathbf{0} \\ \mathbf{G}_1^{-1/2}\tilde{\mathbf{v}}_1 \\ -\mathbf{G}_2^{-1/2}\tilde{\mathbf{z}}_1 \end{array}\right),$$

where $\tilde{\mathbf{u}}_0, \tilde{\mathbf{v}}_0$ are the left and the right singular vectors of $\tilde{\mathbf{B}}_1$ respectively and where $\tilde{\mathbf{v}}_1, \tilde{\mathbf{z}}_1$ are the left and the right singular vectors of $\tilde{\mathbf{B}}_2$ respectively.

References

[1] D. Horak and J. Jost. Spectra of combinatorial Laplace operators on simplicial complexes. *Advances in Mathematics*, 244:303–336, 2013.

[2] B. Eckmann. Harmonische funktionen und randwertaufgaben in einem komplex. *Commentarii Mathematici Helvetici*, 17(1):240–255, 1944.

[3] Lucille Calmon, Michael T. Schaub, and Ginestra Bianconi. Dirac signal processing of higher-order topological signals. *arXiv preprint arXiv:2301.10137*, 2023.
